# Synthesis of Ketjenblack Decorated Pillared Ni(Fe) Metal-Organic Frameworks as Precursor Electrocatalysts for Enhancing the Oxygen Evolution Reaction

**DOI:** 10.3390/molecules28114464

**Published:** 2023-05-31

**Authors:** Thi Hai Yen Beglau, Lars Rademacher, Robert Oestreich, Christoph Janiak

**Affiliations:** Institut für Anorganische Chemie und Strukturchemie, Heinrich-Heine-Universität Düsseldorf, 40204 Düsseldorf, Germany; beglau@hhu.de (T.H.Y.B.); lars.rademacher@hhu.de (L.R.); robert.oestreich@hhu.de (R.O.)

**Keywords:** metal-organic frameworks (MOFs), Ketjenblack, electrocatalysis, oxygen evolution reaction (OER), nickel, iron

## Abstract

Metal-organic frameworks (MOFs) have been investigated with regard to the oxygen evolution reaction (OER) due to their structure diversity, high specific surface area, adjustable pore size, and abundant active sites. However, the poor conductivity of most MOFs restricts this application. Herein, through a facile one-step solvothermal method, the Ni-based pillared metal-organic framework [Ni_2_(BDC)_2_DABCO] (BDC = 1,4-benzenedicarboxylate, DABCO = 1,4-diazabicyclo[2.2.2]octane), its bimetallic nickel-iron form [Ni(Fe)(BDC)_2_DABCO], and their modified Ketjenblack (mKB) composites were synthesized and tested toward OER in an alkaline medium (KOH 1 mol L^−1^). A synergistic effect of the bimetallic nickel-iron MOF and the conductive mKB additive enhanced the catalytic activity of the MOF/mKB composites. All MOF/mKB composite samples (7, 14, 22, and 34 wt.% mKB) indicated much higher OER performances than the MOFs and mKB alone. The Ni-MOF/mKB14 composite (14 wt.% of mKB) demonstrated an overpotential of 294 mV at a current density of 10 mA cm^−2^ and a Tafel slope of 32 mV dec^−1^, which is comparable with commercial RuO_2_, commonly used as a benchmark material for OER. The catalytic performance of Ni(Fe)MOF/mKB14 (0.57 wt.% Fe) was further improved to an overpotential of 279 mV at a current density of 10 mA cm^−2^. The low Tafel slope of 25 mV dec^−1^ as well as a low reaction resistance due to the electrochemical impedance spectroscopy (EIS) measurement confirmed the excellent OER performance of the Ni(Fe)MOF/mKB14 composite. For practical applications, the Ni(Fe)MOF/mKB14 electrocatalyst was impregnated into commercial nickel foam (NF), where overpotentials of 247 and 291 mV at current densities of 10 and 50 mA cm^−2^, respectively, were realized. The activity was maintained for 30 h at the applied current density of 50 mA cm^−2^. More importantly, this work adds to the fundamental understanding of the in situ transformation of Ni(Fe)DMOF into OER-active α/β-Ni(OH)_2_, β/γ-NiOOH, and FeOOH with residual porosity inherited from the MOF structure, as seen by powder X-ray diffractometry and N_2_ sorption analysis. Benefitting from the porosity structure of the MOF precursor, the nickel-iron catalysts outperformed the solely Ni-based catalysts due to their synergistic effects and exhibited superior catalytic activity and long-term stability in OER. In addition, by introducing mKB as a conductive carbon additive in the MOF structure, a homogeneous conductive network was constructed to improve the electronic conductivity of the MOF/mKB composites. The electrocatalytic system consisting of earth-abundant Ni and Fe metals only is attractive for the development of efficient, practical, and economical energy conversion materials for efficient OER activity.

## 1. Introduction

In recent decades, issues caused by the burning of fossil fuels have become a global concern. Therefore, it is important to replace fossil fuels with clean, sustainable, and renewable resources [1,2].

In the past years, water splitting, with the use of renewable electricity for hydrogen generation, seems to be one of the most promising technologies of electrical energy conversion for storage and other uses of H_2_ [3,4]. The electrolytic water-splitting process consists of two half-cell reactions: the hydrogen evolution at the cathode (HER) and the oxygen evolution at the anode (OER). The oxygen evolution reaction consists of three main processes: (i) the adsorption of H_2_O/OH^−^ on the electrocatalyst surface; (ii) the formation of reaction intermediates; (iii) the release of O_2_ molecules. OER passes through a four-electron transfer process coupled with the breaking of the O–H bond and the formation of the O–O bond, which is kinetically slow and requires a high overpotential to overcome the energy barrier. Noble metals such as Ru, Pt, and Ir are highly-active electrocatalysts for the water splitting process [5,6]. However, their commercialization on a large scale is hampered by their scarcity and high cost. Therefore, finding advanced low-cost electrocatalysts that also have long-term stability to replace noble metals for the OER is the primary requirement to enable large-scale electrocatalytic water splitting [7,8,9].

Numerous earth-abundant transition metal (e.g., Fe, Co, Ni, and Cu)-based materials including oxides, hydroxides, nitrides, phosphides, sulfides, and selenides are considered promising toward OER [10,11]. Nickel-based materials have received considerable attention [12]. The optimization of the morphology and porosity of nickel-based oxides and hydroxides [13,14], perovskites [15], nitrides [16], phosphides [17], sulfides [18,19], selenides [20,21], and mixed-metal oxides [22,23] have been published in various extensive works. Subbaraman et al. [24] ranked the electrocatalytic efficiency of 3d M^2+^ (M = Fe, Co, Ni and Mn) ions in the following order: Ni > Co > Fe > Mn. This order is inverse to the strength of the HO–M^2+^ bond [24]. Ni(OH)_2_-based OER catalysts have been identified as good OER catalysts. Stern et al. reported enhanced activity in OER for β-Ni(OH)_2_ nanoparticles with different morphologies, with the low overpotential of 299 mV for Ni(OH)_2_ catalysts [25]. Because of their facile synthesis and excellent OER activities, amorphous metal (oxide) hydroxide materials have consistently outperformed crystalline metal oxides as the preferred catalyst in an alkaline medium [25]. However, it is still a challenge to overcome the low stability of Ni(OH)_2_. Many strategies have been devoted to enhance the stability of Ni(OH)_2_ such as forming the Ni(OH)_2_/NiOOH pair with a small amount of iron [26,27,28,29,30,31] or using a ternary Ni–Co–Fe mixed-metal hydroxide [32,33,34]. The combination of iron into composite materials containing amorphous nickel- or cobalt-based catalysts can be beneficial for electrocatalytic water oxidation [32,33]. The presence of iron promotes the nickel oxidation from +2 to +3, which is an active state and leads to fast reaction kinetics and enhanced conductivity [32,33]. As reported by Yu et al. [30], the electrocatalyst with the optimal Ni/Fe composition of 32/1 performed with the highest activity due to the small reaction resistance and superior intrinsic activity. However, these materials usually have low surface areas and are multi-step synthesis procedures also involving high-temperature treatments. It is still necessary to develop a facile approach to synthesize highly porous and stable nickel hydroxide materials with simple and energy efficient synthesis procedures.

Metal-organic frameworks (MOFs) are porous and crystalline materials constructed from metal ions and multitopic bridging ligands and have been widely studied in many fields such as gas storage and isolation, energy conversion and storage devices as well as electro-catalysts in water splitting [35,36,37,38]. However, most MOFs have poor electrical conductivity and a lack of stability in a strongly acidic or basic aqueous electrolyte [39,40]. However, MOFs are promising precursor candidates toward OER catalysts based on their high surface area and porosity [41]. For example, Ni-MOFs decompose in an alkaline medium (e.g., 1 mol L^−1^ KOH) into a mixture of α/β-Ni(OH)_2_, α/β-NiOOH, and γ-NiOOH, which appear to be mainly responsible for the observed OER activity [42,43,44,45,46]. MOFs are often mixed with conductive carbon materials such as graphene [47], graphene oxide (GO) [48], reduced graphene oxide (RGO) [49], or carbon nanotubes (CNT) [50,51] to overcome the low electrical conductivity of MOFs. These carbon materials not only elevate the electrical conductivity of MOFs, but also give a homogenous dispersion to enhance the electrocatalytic performances of the formed catalyst under highly alkaline conditions. Ketjenblack carbon (KB) has a high surface area (∼1300 m^2^ g^−1^), low cost, excellent charge-transport properties, and superior chemical stability. Currently, there are various reports on MOFs and nanoparticles with Ketjenblack carbon that were used as catalysts for OER [41,44,52,53,54,55]. For instance, Sondermann et al. [44] showed that the bimetallic Ni_10_Co-BTC/KB composite gave an overpotential of 344 mV at a current density of 10 mA cm^−2^ and a Tafel slope of 47 mV dec^−1^ in 1 mol L^−1^ KOH. Öztürk et al. [55] reported Ni(Fe)-MOF-74 with KB for OER in an alkaline media. The required overpotential to achieve a current density of 10 mA cm^−2^ was only 274 mV and the Tafel slope was 40 mV dec^−1^.

**Scheme 1:**
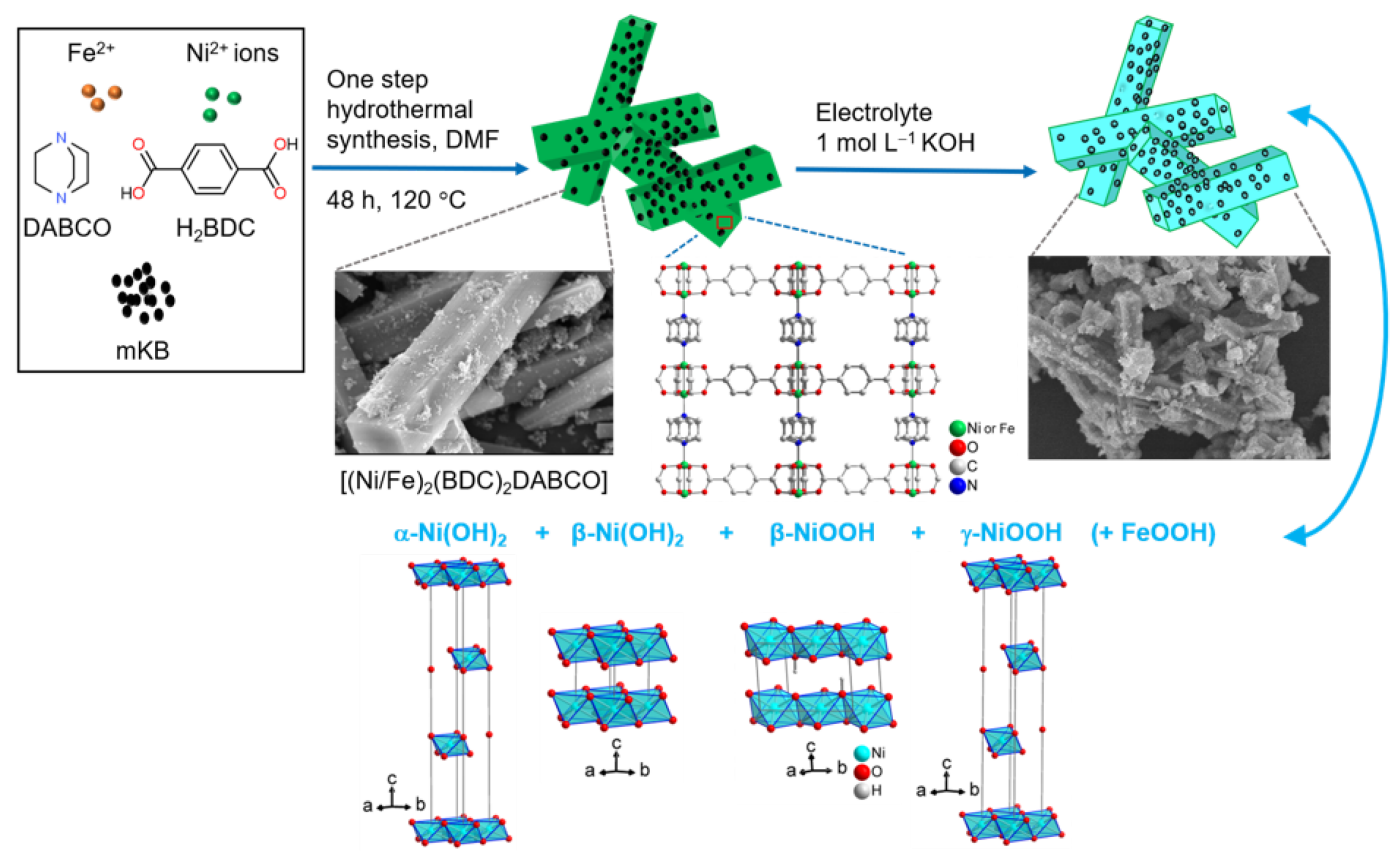
Schematic illustration of the process used for the synthesis of the NiDMOF/mKB and Ni(Fe)DMOF/mKB composites and the transformation of the MOF into metal oxide-hydroxides with the retention of the MOF morphology. For the nickel (oxy)hydroxides, the unit cells of the crystal structures are given in polyhedral mode.

In this work, we report on the synthesis of the Ni-based pillared MOF [Ni_2_(BDC)_2_DABCO], (BDC = 1,4 benzenedicarbonxylate, DABCO = 1,4 diazabicyclo [2.2.2] octane), known as NiDMOF [56] as well as with the addition of iron as Ni(Fe)DMOF and as a composite with a modified Ketjenblack carbon (mKB) through a facile one-pot synthesis method (Scheme 1). NiDMOF consists of {Ni_2_(O_2_C-)_4_} paddle-wheel clusters connected by BDC linkers to form two dimensional layers that are pillared by DABCO ligands to a three-dimensional structure (Figure S1, Supplementary Materials). The pretreatment of KB with nitric acid adds hydrophilic oxygen-containing groups that are beneficial to improving the wettability and interfacial area, promoting electrolyte accessibility. Good wettability strengthens the ion-accessible surface area, promotes charge transportation between the electrolyte and electrode, and enables effective electrical integration to minimize the ohmic losses, leading to the improved OER activity [57,58]. The Ni(Fe)DMOF/mKB materials were employed as precursors to OER electrocatalysts in a KOH electrolyte. Stability tests of the MOF in KOH and post-mortem analyses showed the transformation of the precursor into α/β-Ni(OH)_2_ and β/γ-NiOOH (Scheme 1), albeit with the retention of the MOF morphology.

## 2. Results and Discussion

### 2.1. Characterization

Scheme 1 illustrates the procedure to prepare Ni(Fe)DMOF and its mKB composite. Nickel nitrate, together with iron acetate, benzene-1,4-dicarboxylic acid (H_2_BDC), and 1,4 diazabicyclo[2.2.2]octane (DABCO), afford the products [Ni_2_(BDC)_2_DABCO] and [(Ni/Fe)_2_(BDC)_2_DABCO], abbreviated as NiDMOF and Ni(Fe)DMOF, respectively. In the presence of mKB, the composites NiDMOF/mKBx with different mKB fractions x = 7, 14, 22, and 34 wt.%. or Ni(Fe)DMOF)/mKB14 were obtained by using a one-step solvothermal reaction at 120 °C in DMF for 48 h. The synthesis of Ni(Fe)DMOF was carried out with a targeted molar Ni to Fe ratio of 32:1. A molar Ni to Fe ratio of 30:1 was achieved in the synthesized Ni(Fe)DMOF, as determined from atomic absorption spectroscopy (AAS) and SEM-EDX (Tables S2 and S3, Figure S9, Supplementary Materials). The weight fractions of mKB in the MOF/mKBx composites were about 7, 14, 22, and 34 wt.%, calculated from the MOF content, which was derived from the nickel amount by the atomic absorption spectroscopy (AAS) data (Table S2, Supplementary Materials).

**Figure 1:**
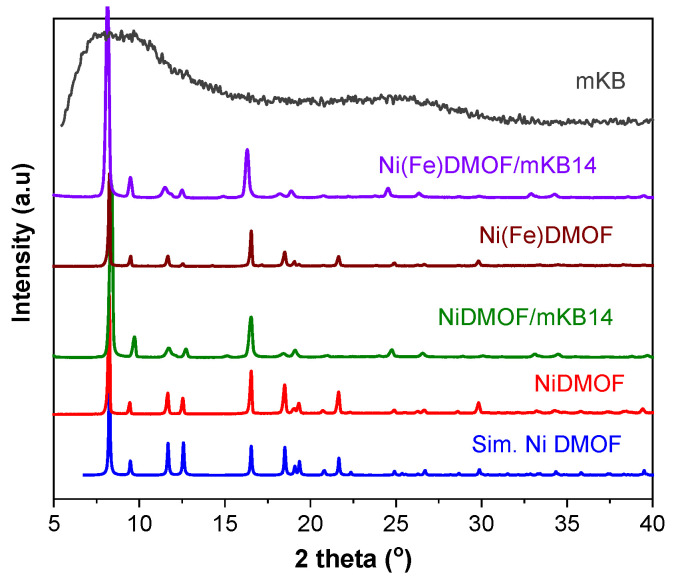
The PXRD patterns of NiDMOF, NiDMOF/mKB14, Ni(Fe)DMOF, Ni(Fe)DMOF/mKB14, and mKB. The simulated PXRD pattern of NiDMOF was obtained from CCDC no. 802892. The first five reflexes at 2θ = 8.2°, 9.4°, 11.7°, 12.4°, and 16.6° corresponded to the 100, 001, 110, 101 and 200 planes, respectively.

As seen in the literature, the electric conductivity of the samples increases significantly by adding mKB (or another carbon material), thereby improving the excellent OER activities and cycling stability [59,60,61,62,63,64]. However, since mKB has almost no electrochemical activity, increasing the fraction of mKB would lead to lower energy density [65]. Only a small amount of mKB is needed to enhance the rate of OER performance as the electrons can also move due to incoherent jumps between neighboring locations. Furthermore, we will see later that the MOF is only the precursor. The active species are better conducting Ni–Fe oxide-hydroxides, for which the mKB then serves as a contact to the glassy carbon or nickel foam electrode. The experimental powder X-ray diffractogram (PXRD) of the MOF product was positively matched to the reported structure of [Ni_2_(BDC)_2_DABCO]·(DMF)_4_·(H_2_O)_1.5_ (NiDMOF) (Figure 1a and Scheme S1) [56]. The PXRD also demonstrated that the presence of mKB influenced the crystallinity of the NiDMOF structure only at high mKB content in NiDMOF/mKB34, where peak broadening occurs (Figure 1 and Figure S2, Supplementary Materials). This is due to the formation of a larger amount of smaller MOF crystallites, also evident from the scanning electron microscopy images (Figure S7, Supplementary Materials). In Figure 1, the main PXRD reflection of Ni(Fe)DMOF/mKB14 shifted to lower degrees compared with other counterparts, indicating an enlarged structural unit and the existence of a tensile strain. The crystal tensile strain will help to adsorb OH^–^ reactants to improve the OER performance [66,67]. The functional groups –OH, C–O, and –COOH of mKB can serve as crystallization points, leading to more crystal seeds, which subsequently do not grow into large crystals [68,69,70]. Furthermore, the ratio of the 001/100 reflex intensities increases in the NiDMOF/mKBx composites with mKB content, indicating that in the presence of mKB, the DMOF crystallites may become more oriented along the [001] direction of the NiDMOF structure (Figure S1, Supplementary Materials).

**Figure 2:**
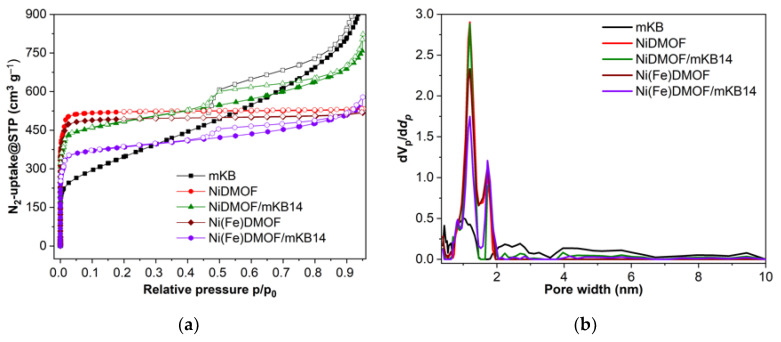
(**a**) Nitrogen sorption isotherms at 77 K (filled symbols adsorption; empty symbols desorption) and (**b**) the pore size distribution of NiDMOF, NiDMOF/mKB14, Ni(Fe)DMOF, Ni(Fe)DMOF/mKB14, and mKB.

The electrochemical performance is strongly correlated to the surface area, porosity and pore size distribution of electrode materials [71]. To explore these properties, nitrogen sorption measurements were performed at 77 K and gave the expected Type I isotherms [72] for microporous NiDMOF and Ni(Fe)DMOF (Figure 2a and Figure S6a). The specific BET surface area and total pore volume of NiDMOF was 2104 m^2^ g^−1^ and 0.82 cm^3^ g^−1^, respectively, in good agreement with the literature values (2050 m^2^ g^−1^, 0.80 cm^3^ g^−1^) (Table 1) [73]. Ni(Fe)DMOF also showed a similar high BET surface area and total pore volume with 1942 m^2^ g^−1^ and 0.80 cm^3^ g^−1^, respectively. The BET surface area and total pore volume of modified Ketjenblack (mKB) were 1234 m^2^ g^−1^ and 1.50 cm^3^ g^−1^, identical to the original Ketjenblack carbon (KB) (Table 1 and Figure S5a,b, Supplementary Materials). For the DMOF/mKBx composites, the desorption branch additionally displayed a Type H4 hysteresis [72], as seen in the isotherm of the mesoporous mKB carbon itself (Figure 2a and Figure S5c,d). The experimental surface area and pore volume parameters of the composites NiDMOF/mKBx (x = 7, 14, 22, 34 wt.%) and Ni(Fe)DMOF/mKB14 were somewhat lower than those estimated from the mass-weighted values of the neat MOF and mKB components (Table 1). This reduction in the BET value of composites was also observed for similar work and indicates that mutual pore blocking effects occur [55]. The NiDMOF can grow into the mKB pores or on the mKB surface, and therefore, access to the mKB and NiDMOF pores becomes restricted. The SEM images for NiDMOF/mKBx with a higher amount of mKB (x ≥ 22 wt.% mKB) illustrate the surface coverage of the NiDMOF rods with mKB particles (Figure S7, Supplementary Materials). At the same time, the combination of mKB with MOFs can increase the total (micro-meso)pore volume in the composites above the estimated value. The composite NiDMOF/mKB14 exhibited an experimental total pore volume of 1.18 cm^3^ g^−1^, considerably higher than the estimate of 0.92 cm^3^ g^−1^ for a physical mixture of 86 wt.% NiDMOF and 14 wt.% mKB. This pore volume of 1.18 cm^3^ g^−1^ was also the maximum among the prepared DMOF/mKB composites.

The synthesized NiDMOF and Ni(Fe)DMOF samples exhibited micropores with widths of about 1.2 and 1.7 nm (Figure 2b and Figure S6d). In the NiDMOF/mKBx and Ni(Fe)DMOF/mKB14 composites, there were additional mesopores above 2 nm from the mKB component. The increased pore volume beyond the estimate could be traced to interparticle mesopores above 5 nm (Table 1 and Figure S6a). Open microporous channels can provide active sites for the electrochemical reaction and open mesoporous channels will improve the diffusion rate of electrolyte ions [74].

**Figure 3:**
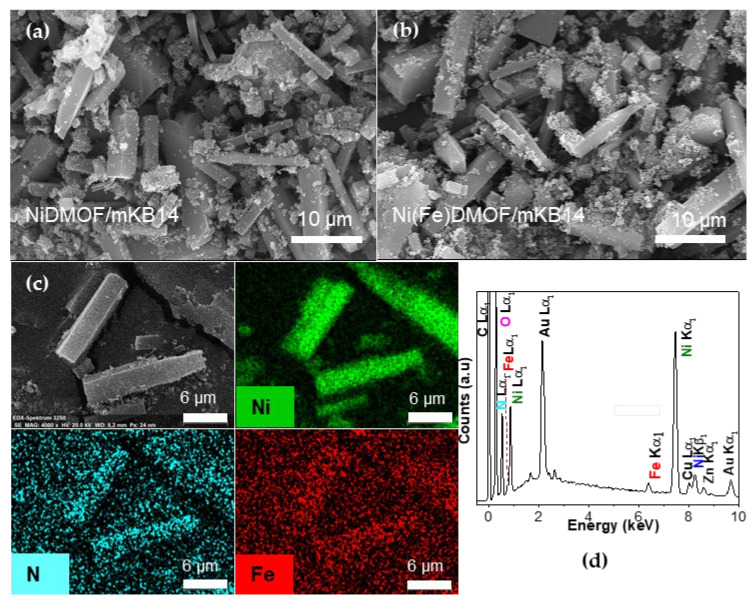
SEM images of (**a**) NiDMOF/mKB14, (**b**) Ni(Fe)DMOF/mKB14, (**c**) EDX scanning element mapping for nickel, nitrogen and iron for Ni(Fe)DMOF/mKB14, and (**d**) the EDX spectrum with the composition for the selected mapping area of Ni(Fe)DMOF/mKB14.

Scanning electron microscopy (SEM) of the MOFs and the composites showed rod or block microcrystals of the NiDMOF with an increasing amount of fine grains of mKB from NiDMOF/mKB7 to NiDMOF/mKB34 (Figure 3 and Figure S6, Supplementary Materials). The observed NiDMOF morphology was in a good agreement with the reported one [75]. The element mapping from the SEM-energy-dispersive X-ray spectroscopy (SEM-EDX) (Figure 3c and Figure S8, Supplementary Materials) demonstrated that in Ni(Fe)DMOF, the iron is homogeneously distributed in the MOF microcrystals.

**Figure 4:**
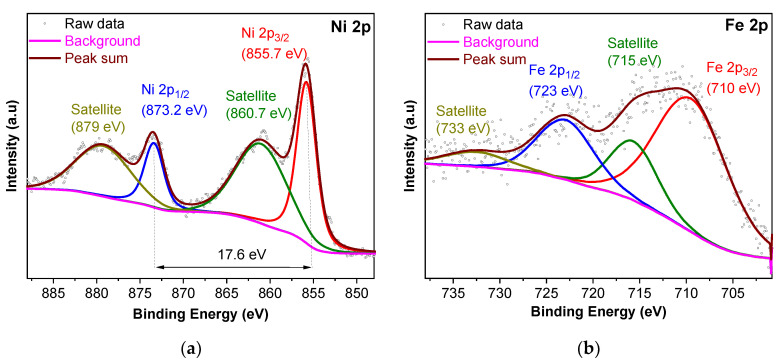
High-resolution XPS spectra of (**a**) Ni 2p and (**b**) Fe 2p of Ni(Fe)DMOF/mKB14.

The X-ray photoelectron spectroscopy (XPS) survey spectrum of Ni(Fe)DMOF/mKB14 confirmed the presence of all the elements (Ni, Fe, C, N, and O) of the synthesized material (Figure S10, Supplementary Materials for XPS of NiDMOF and Ni(Fe)DMOF). The high-resolution spectrum of Ni 2p (Figure 4a) showed two characteristic peaks at 855.7 and 873.2 eV, which were ascribed to Ni^2+^ 2p_3/2_ and Ni^2+^ 2p_1/2_, respectively, and two expected satellite peaks were located at 860.7 and 879 eV [25,76,77,78]. A spin-orbit coupling energy difference between the 2p_3/2_ and 2p_1/2_ binding energy of 17.6 eV supports the assignment of the +2 oxidation state [25,79]. Furthermore, in the Fe 2p spectrum, the peaks at 710 and 723 eV (Figure 4b) confirmed the +2 oxidation state of Fe [80,81]. The 2p^3/2^ spectrum range was 710 to 720 eV including a satellite peak at 715 eV, while the 2p_1/2_ spectrum range was from 721 to 735 eV with a satellite peak at 733 eV. It should be noted that the Fe 2p spectral background had a contribution from a Ni_LMM_ Auger peak [82]. More detailed information on the high-resolution XPS of C 1s, O 1s, N 1s, and Fe 3p of Ni(Fe)DMOF/mKB14, Ni 2p and Fe 2p of Ni(Fe)DMOF, Ni 2p of NiDMOF is given in the Supplementary Materials (Figures S11 and S12).

### 2.2. Electrochemical Properties

**Figure 5:**
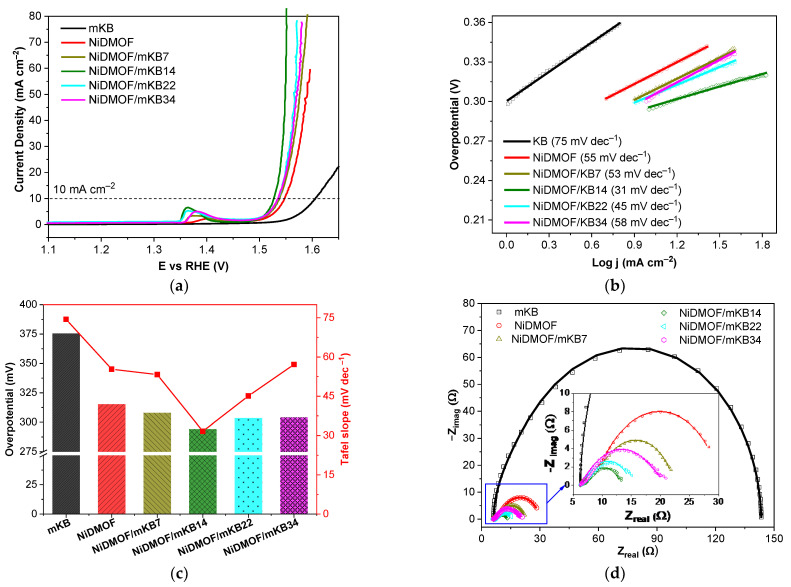
(**a**) Polarization curves (LSV) operated at a scan rate of 5 mV s^−1^ with the iR correction. (**b**) Tafel plots, (**c**) corresponding overpotentials (columns) and Tafel slopes (red dots) at 10 mA cm^−2^, (**d**) raw data of Nyquist plots at 1.5 V vs. RHE (points) and fitting to an equivalent circuit model (Figure S16, Supplementary Materials) (solid line) from the EIS test of mKB, NiDMOF, NiDMOF/mKB7, NiDMOF/mKB14, NiDMOF/mKB22, and NiDMOF/mKB34.

The OER performance of different samples was evaluated by using a glassy carbon rotating disk electrode (GC-RDE) under alkaline conditions (1 mol L^−1^ KOH). OER polarization curves were collected from the linear sweep voltammetry (LSV) measurements at a sweep rate of 5 mV s^−1^. As shown in the initial LSV curves (Figure 5a), the NiDMOF/mKBx composites were more efficient for OER activity than pristine NiDMOF and mKB alone at a current density of 10 mA cm^−2^. Thereby, it is worth noting that the OER activity of the NiDMOF sample is already as good as the commercial RuO_2_ benchmark sample. Among all NiDMOF/mKBx composites (x = 7, 14, 22, 34 wt.% mKB), NiDMOF/mKB14 exhibited the best electrocatalytic activity with the smallest overpotential of 294 mV (vs. RHE at 1.23 V) to achieve a current density of 10 mA cm^−2^, which was much lower than the overpotential of mKB (375 mV), NiDMOF (315 mV), NiDMOF/mKB7 (308 mV), NiDMOF/mKB22 (302 mV), and NiDMOF/mKB34 (304 mV), and competes with the performance of the benchmark material RuO_2_ (317 mV). Thereby, the overpotential of RuO_2_ is in good accordance with the literature [83]. A low overpotential means a lower demand of energy for the oxygen evolution reaction. The kinetics on the GCE toward NiDMOF and its DMOF/mKBx composites were described on the basis of the Tafel equation. The OER kinetic parameters of the samples were analyzed by the Tafel equation (η = a + b log(*j*)), which is used to determine the reaction mechanism and the kinetics [84]. The Tafel slope indicates how much one has to increase the overpotential to increase the reaction rate by a factor of ten. It describes the influence of the overpotential on the steady-state current density and is an important parameter for the evaluation of OER kinetics. The Tafel slope was calculated from corresponding LSV plots to obtain a quantitative idea about the electrocatalytic performance. Krasil’shchikov’s OER mechanism is one of the established mechanisms, which is described by Reactions (3)–(6) with the corresponding Tafel slopes *b* [44,85,86].

M + OH^−^ ⇆ MOH + e^−^, *b* = 120 mV dec^−1^ (2)

MOH + OH^−^ ⇆ MO^−^ + H_2_O, *b* = 60 mV dec^−1^ (3)

MO^−^ → MO + e^−^, *b* = 45 mV dec^−1^ (4)

2MO → 2M + O_2_, *b* = 19 mV dec^−1^ (5)

The value of the Tafel slope in NiDMOF (55 mV dec^−1^) is in between Reactions (3) and (4), and indicates that the deprotonation of a metal hydroxide (3) and the oxidation of a metal oxide species (4) could occur together as rate-determining steps [86,87]. The corresponding Tafel curves derived from Figure 5b show the Tafel slopes for NiDMOF/mKBx composites (7, 14, 22, 34 wt.% mKB) with 53, 32, 45, and 51 mV dec^−1^, respectively. Thus, integrating mKB with NiDMOF can enhance the kinetics of the catalyst. For the 14 and 22 wt.% composites, there was also a change in the rate-determining step toward Reaction (4) for 22 wt.% (slope of 45 mV dec^−1^) and toward Reaction (5) of the evolution of O_2_ for 14 wt.% (slope of 32 mV dec^−1^). The NiDMOF/mKB14 composite had the smallest Tafel slope (32 mV dec^−1^), confirming that the interaction between NiDMOF and 14 wt.% of mKB in the composite gives the most proficient material.

It is acknowledged that due to the surface structure reconstruction of Ni-based catalysts, an activation occurs during the water oxidation process [30,44]. In order to understand the electrocatalytic behavior and the activation process, 100 cyclic voltammetry (CV) scans were applied. With mKB present in the composites, the oxidation peak at 1.35–1.45 (V vs. RHE) became more noticeable and shifted into a positive direction, indicating the synergetic effect of mKB and NiDMOF on the oxidation of Ni^+2^ to Ni^+3^ (Figure S13, Supplementary Materials). As seen in Figure S14, the CV curves of all samples after 100 CVs showed that NiDMOF/mKB14 provided the most active catalyst, while mKB alone exhibited worse OER activity due to carbon corrosion in alkaline conditions [88]. The mKB additive led to the electrical conductivity between the active Ni sites and the GCE, and thereby enhanced the electrochemical activity [89,90]. However, an excessive amount of mKB lowered the carbon dispersion due to agglomeration from the π–π interaction between the carbon particles, which would lead to lower energy density for OER [65,91,92].

Furthermore, electrochemical impedance spectroscopy (EIS) was carried out to assess the kinetics of the electrode reaction. The corresponding Nyquist plots (Figure 5d) obtained in a frequency range from 0.01 to 100 kHz with an AC potential amplitude of 5 mV at 1.5 V (RHE) were fitted to the simplified equivalent circuit by Doyle et al. for OER (Figure S16, Supplementary Materials) [30,93,94,95]. The kinetics of the interfacial charge transfer reaction corresponded to the resistive components R_s_ and R_p_ in terms of the electrocatalytic capabilities of the oxide layer. In particular, the polarization resistance (R_p_) is considered as a total charge transfer resistance across multiple steps of OER, while R_s_ is related to the rate of production of one or more surface intermediates [30,93,94,95]. More detailed information concerning the circuit parameters is given in Figure S16. From the shape of the semicircles in the Nyquist plots, it can be seen that NiDMOF and the NiDMOF/mKBx composites follow a similar trend. Smaller semicircles are displayed by the NiDMOF/mKBx composites, which reflect smaller resistances at high frequency compared with both the pure NiDMOF and mKB, which illustrates that the kinetic performances of the neat MOF materials were improved by the integration of mKB. The low polarization resistance R_p_ (4Ω) and resistance adsorption components of the reaction intermediate R_s_ (2 Ω) for NiDMOF/mKB14 indicate a superior charge transfer rate and the easier formation of active species for OER, respectively, which contribute to its highest catalytic activity among the composites (Table S4, Supplementary Materials). Thus, mKB lowers the overall resistances, which is considered to be a key factor in enhancing the electrochemical performance of MOF-based composites.

**Figure 6:**
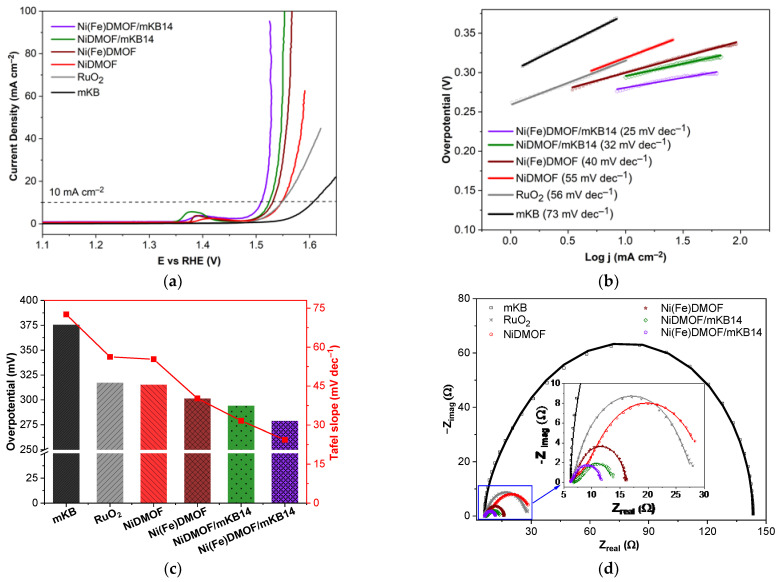
(**a**) Polarization curves (LSV) operated at a scan rate of 5 mV s^−1^ with iR correction, (**b**) Tafel plots, (**c**) corresponding overpotentials (columns) and Tafel slopes (red dots) at 10 mA cm^−2^, (**d**) raw data of Nyquist plots at 1.5 V vs. RHE (points) and the fitting data of an equivalent circuit model (Figure S16, Supplementary Materials) (solid line) from the EIS test of mKB, RuO_2_, NiDMOF, Ni(Fe)DMOF, NiDMOF/mKB14, and Ni(Fe)DMOF/mKB14.

It has been intensively studied that Fe can be advantageously incorporated into Ni-based catalysts. With a small amount of Fe, the catalytic activity toward OER can be significantly improved [26,28,30,31,33,44,55]. In order to investigate the effect of Fe on the electrocatalytic NiDMOF performance, a bimetallic Ni(Fe)DMOF and the Ni(Fe)DMOF/mKB14 composite were prepared and measured under the same conditions. The presence of a small amount of Fe at a Ni:Fe ratio of ~30:1 can significantly enhance the OER performance. The Ni(Fe)DMOF without mKB already exhibited an enhanced OER performance with a much lower overpotential of 301 mV at the current density of 10 mA cm^–2^, which was much smaller compared to the commercial RuO_2_ benchmark (317 mV) and pristine NiDMOF (315 mV). The composite of Ni(Fe)DMOF with 14 wt.% mKB further enhanced the OER activity, giving the smallest overpotential of only 279 mV among the materials reported here (Figure 6a,c). These results strongly demonstrate that Fe plays a key role in improving the OER activities of pure NiDMOF and its composites. As shown in Figure 6b,c, the Tafel slope calculated from the corresponding LSV curve of bimetallic Ni(Fe)DMOF was 40 mV dec^−1^, which is in agreement with a thin-film of Ni–Fe oxide [96] and outperformed the monometallic NiDMOF (55 mV dec^−1^). Ni(Fe)DMOF/mKB14 presented the lowest Tafel slope (25 mV dec^−1^) compared to RuO_2_ (56 mV dec^−1^) and all DMOFs and composites investigated here (Table S4, Supplementary Materials), reflecting the fastest kinetics. The results indicate a change in the mechanism where the rate-determining step becomes the evolution of O_2_ (Reaction (5)). Furthermore, the Ni(Fe)DMOF/mKB14 composite stands out as one of the best in the recently reported most advanced Ni-based electrocatalysts with both its low overpotential (279 mV) and Tafel slope (25 mV dec^−1^) (Table S4, Supplementary Materials). A lower overpotential was reported for Ni(Fe)-MOF-74/KB (48 wt.% KB) (274 mV) [55], Ni(Fe)(OH)_2_/KB (47 wt.% KB) (265 mV) [55], and Fe-doped HXP@NC800 (266 mV) [97] (all at 10 mA cm^−2^), albeit with larger Tafel slopes of 40, 55, and 49 mV dec^−1^, respectively. From Figure S15, it can be seen that even after 1000 CVs, the overpotential of the catalysts only slightly changed from 301 mV to 318 mV for the Ni(Fe)DMOF precursor and from 279 mV to 285 mV for Ni(Fe)DMOF/mKB14 (at 10 mA cm^−2^), indicating their long-term activity. Furthermore, electrochemical impedance spectrometry (EIS) measurements were carried out to understand the charge-transfer kinetics during the OER process among the different samples. As shown by the Nyquist plots (Figure 6d) and fitted data of the equivalent circuit model in Figure S16, the resistances of the charge-transfer and adsorption components of the reaction intermediates of Ni(Fe)DMOF/mKB14 (R_p_ 4 Ω and R_s_ 2 Ω) was as low as NiDMOF/mKB14 (R_p_ 4 Ω and R_s_ 2 Ω), that is, lower than that of RuO_2_ (R_p_ 23 Ω and R_s_ 4 Ω), NiDMOF (R_p_ 21 Ω and R_s_ 7 Ω), and Ni(Fe)DMOF (R_p_ 6 Ω and R_s_ 4 Ω) (Table S4, Supplementary Materials). The polarization resistance (R_p_) of Ni(Fe)DMOF/KB14 was much smaller than that of Ni_32_Fe oxide (17.1 Ω) [30], IrO_2_ (~5 Ω) [86], NiCo_2_O_4_ (~8 Ω) [86], Ni_1.7_Co_1.3_O_4_ (~6 Ω) [98], H-SCF_0.55_ (~58 Ω) [99] (all data fitted with the same equivalent circuit model). The catalyst from the Ni(Fe)DMOF/mKB14 precursor presented the smallest charge-transfer resistance, which indicates its faster electron transport kinetics and intrinsic excellent electrical conductivity, well in accordance with its lowest overpotential. This result is in good agreement with the identified higher catalytic activity of Ni(Fe)-MOFs compared to analogous Ni-MOFs [30,55,100,101]. In particular, the presence of iron promotes the oxidation of nickel from +2 to +3, the latter seen as the active state of fast reaction kinetics and enhanced conductivity.

**Figure 7:**
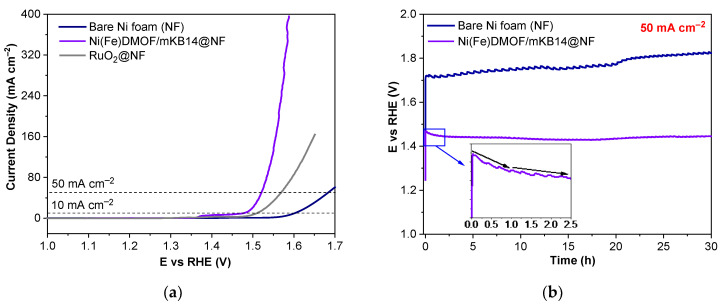
(**a**) Linear sweep voltammetry (LSV) curves at a scan rate of 5 mV s^−1^ with iR correction. (**b**) Chronopotentiometric curves for 30 h at a current density of 50 mA cm^−2^ of the NiFe-DMOF/mKB14 loaded on nickel foam (NF) and bare NF for comparison in a 1.0 mol L^−1^ KOH solution.

Loading a catalyst on a porous Ni foam (NF) electrode can further improve the reaction rate of OER [102]. Because of its metallic conductivity and 3D macroporous structure, NF provides a large surface area and facilitates mass transport during OER [30]. The catalyst ink was loaded by the drop-casting method on the surface of NF with a good distribution of the ink layer on the scaffold of the NF. The sample Ni(Fe)DMOF/mKB14, which showed the best OER activity on the glassy carbon RDE, was deposited on NF (1 cm^2^) (Figure S17, Supplementary Materials). The LSV curves in Figure 7a showed that the activity of Ni(Fe)DMOF/mKB14@NF was much higher than the pure NF substrate, reaching current densities of 10 and 50 mA cm^−2^ with overpotentials of 247 and 291 mV, respectively. A practical current density of 400 mA cm^−2^ could be delivered for Ni(Fe)DMOF/mKB14@NF at a low overpotential of 381 mV. This performance was better than the benchmark RuO_2_@NF as an OER catalyst with overpotentials of 278 and 340 mV at current densities of 10 and 50 mA cm^−2^, respectively (Figure 7a), the latter being in good accordance with the literature [103]. The pure NF electrode showed a much lower OER activity with an overpotential of 370 mV at 10 mA cm^−2^, similar to the literature [103].

The stability of an electrocatalyst determined by chronoamperometry is a key parameter to evaluate the practical application of a material. A long-term stability measurement was carried out by applying a potential to reach a constant current density of 50 mA cm^−2^ for 30 h (Figure 7b). The applied potential could be gradually decreased during the first 2.5 h, indicating that the Ni–Fe catalyst was activated under the anodic potential in 1 mol L^−1^ KOH electrolyte. After this time, the potential remained nearly unchanged during the rest of the 30 h measurement. In contrast, the bare NF electrode showed an increasing potential over time for delivering a current density of 50 mA cm^−2^ due to the oxidation of nickel, which occurs at the surface of the foam but can also reach its subsurface during electrolysis under a high current density [30,104]. Pure NF also necessitates a much higher potential at the current density of 50 mA cm^−2^ than Ni(Fe)DMOF/mKB14@NF during the 30 h of the chronoamperometry test. The catalyst derived from Ni(Fe)DMOF/mKB14 does not only have a robust stability, but also an outstanding activity to deliver a high current density, which demonstrates that the Ni(Fe)DMOF/mKB14 precursor has the potential to serve as a good OER catalyst for practical applications.

**Figure 8:**
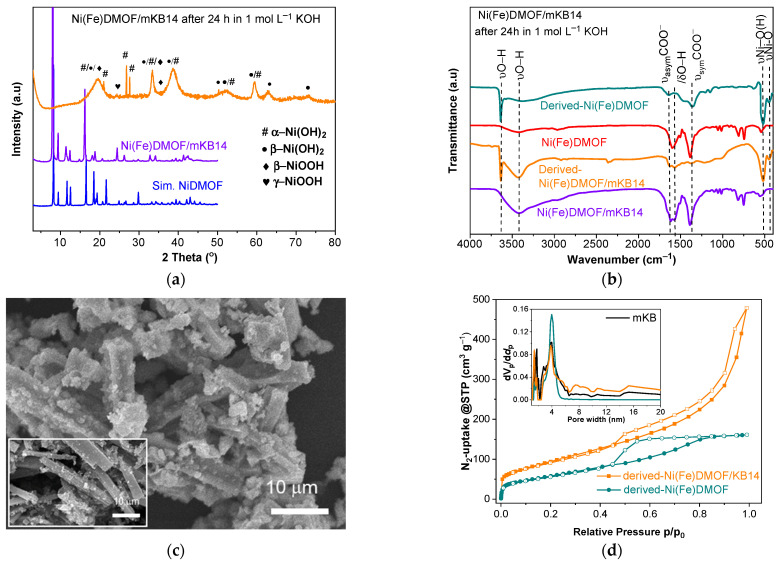
(**a**) PXRD patterns of the experimental Ni(Fe)DMOF/mKB14 and derived-Ni(Fe)DMOF/mKB14 after 24 h in 1 mol L^−1^ KOH and simulated NiDMOF (CCDC Nr. 802892). Reflections from β–Ni(OH)_2_ (●, ICDD: 14-0117), α–Ni(OH)_2_ (#, ICDD: 38-0715), β–NiOOH (◆, ICDD: 06-0141), and γ–NiOOH (♥, ICDD: 06-0075). (**b**) FTIR of Ni(Fe)DMOF and Ni(Fe)DMOF/mKB14 and its derived products in 1 mol L^−1^ KOH after 24 h. (**c**) SEM images of the derived-Ni(Fe)DMOF/mKB14 material (large image) and neat Ni(Fe)DMOF/mKB14 (small image). (**d**) Nitrogen sorption isotherms at 77 K (filled symbols adsorption, empty symbols desorption isotherm) and pore size distribution (small graph) of derived-Ni(Fe)DMOF and derived-Ni(Fe)DMOF/mKB14 after 24 h in 1 mol L^−1^ KOH.

In order to understand the transformation of the precursors Ni(Fe)DMOF and Ni(Fe)DMOF/mKB14 in the alkaline electrolyte (1 mol L^−1^ KOH), we reacted macroscopic amounts of these samples in 1 mol L^−1^ KOH, followed by PXRD, FTIR, SEM, and N_2_ sorption measurements to mimic post-mortem experiments of the minuscule electrode materials. The derived materials were collected by filtration after soaking Ni(Fe)DMOF and Ni(Fe)DMOF/mKB14 in 1 mol L^−1^ KOH electrolyte for 24 h and were dried for at least 12 h at 120 °C under vacuum (<10^−2^ mbar). The PXRD pattern of the derived-Ni(Fe)DMOF/mKB14 (Figure 8a) showed the disappearance of the crystalline MOF and suggests the formation of α/β–Ni(OH)_2_, β–NiOOH, and γ–NiOOH from the reflections assigned in Figure 8a based on the patterns of nickel(II) hydroxides and nickel(III) oxide-hydroxides. This implies the transformation from Ni(Fe)DMOF/mKB14 to nickel and iron oxide-hydroxides under a strong alkaline environment (1 mol L^−1^ KOH) [105], which has also been observed by other researchers [106,107,108,109,110]. The FTIR of the derived-materials (Figure 8b and Table S1) showed two broad bands at ~3400 and ~3600 cm^−1^, which corresponded to the stretching vibrations of the adsorbed water molecules and to the O–H stretching vibrations, as in Ni(OH)_2_. At the same time, bands due to the asymmetric vibration ν_asym_COO^−^ and the bending vibrations of the adsorbed water molecules of the δO–H hydroxyl groups of the BDC phenyl ring at ~1600 and ~1570 cm^−1^, along with ν_sym_COO^−^ at ~1350 cm^−1^, decreased in intensity. New strong bands at 516 and 450 cm^−1^ were attributed to the Ni–OH bending vibrations in Ni(OH)_2_ and the oxide-hydroxides [111]. Interestingly, the morphology of Ni(Fe)DMOF/mKB14, as seen with SEM (Figure 8c), did not change profoundly. The rod shape of Ni(Fe)DMOF was largely retained in the alkaline treatment. Accordingly, N_2_ physisorption measurements at 77 K revealed a residual BET surface area and micro-mesoporosity in the derived-Ni(Fe)DMOF and derived-Ni(Fe)DMOF/mKB14 (Figure 8d). The derived-Ni(Fe)DMOF displayed a Type IV isotherm with H2(b) hysteresis (associated with pore blocking) [72] and a BET surface area of 222 m^2^ g^−1^. The adsorption isotherm of the derived-Ni(Fe)DMOF/mKB14 lacked the final saturation plateau of a Type IV isotherm and appeared like a Type II isotherm with an H_3_ hysteresis, which would be given by a largely macroporous adsorbent. The pore size distribution curves showed mostly mesopores (>2 nm) for the derived-Ni(Fe)DMOF and the coexistence of micropores and mesopores for the derived-Ni(Fe)DMOF/mKB14 with micropores and small mesopores due to the mKB part (the macropore size is not given by N_2_ sorption). The BET surface area of the derived-Ni(Fe)DMOF/mKB14 was 352 m^2^ g^−1^ with the increase over derived-Ni(Fe)DMOF coming from the mKB portion. Overall, a hierarchical porous nature of derived-Ni(Fe)DMOF/mKB14 was evidenced by its isotherm and hysteresis shape and the analysis of the pore size distribution. The decomposition of the Ni-MOF precursor and transformation to Ni(OH)_2_/NiOOH was also observed in other work [112,113,114]. The OER activity of the Ni(Fe)DMOF precursor is due to the in situ formation of α/β-Ni(OH)_2_/FeOOH, followed by β/γ–NiOOH under the oxidizing anodic potential, which favors the kinetics of OER [43,44,115,116,117]. Furthermore, the degradation of the micro-mesoporosity of Ni(Fe)DMOF in an alkaline environment provides a large number of accessible active Ni (and promoter Fe) sites in the still porous hydroxides, which show better electrochemical activities and longer cycle numbers than crystalline and dense metal oxide catalysts [42,43,118,119,120,121]. In addition, a suitable amount of mKB (14 wt.%) in the composites provides electrical conductivity with its small particle size, mixes well with the Ni active sites, and avoids the carbon corrosion effect of carbon materials [59,60,61,62,63,64,65,66].

## 3. Materials and Methods

### 3.1. Materials

Nickel(II) nitrate hexahydrate (Ni(NO_3_)_2_⋅6H_2_O, Merck, Darmstadt, Germany), iron(II) acetate (Fe(OAc)_2_, 99.99%, Sigma-Aldrich, St. Loius, MO, USA), ruthenium(IV) oxide (RuO_2_, 99.9%, Sigma-Aldrich, St. Loius, MO, USA), benzene-1,4-dicarboxylic acid (H_2_BDC, 98%, Alfa Aesar, Karlsruhe, Deutschland), 1,4-diazabicyclo[2.2.2]octane (DABCO, 98%, Sigma-Aldrich, St. Loius, MO, USA), perfluorinated resin solution containing Nafion™ 1100W (5 wt.% in lower aliphatic alcohols and water, Sigma-Aldrich, St. Loius, MO, USA), potassium hydroxide solution (KOH, 1 mol L^−1^, Carl Roth, Karlsruhe, Germany), nitric acid (HNO_3_, 65%, Sigma-Aldrich, Darmstadt, Germany), *N*,*N*-dimethylformamide (DMF, 99.99 %, Fisher, Schwerte, Germany), Ketjenblack EC 600 JD (AkzoNobel, Amsterdam, The Netherlands), and methanol (MeOH, 99.98 %, Sigma-Aldrich, Darmstadt, Germany) were purchased and used without further purification. Nickel foam (NF) was obtained from Racemat BV (Dodewaard, The Netherlands), cleaned with 1 mol L^−1^ HCl solution in an ultrasound bath for 5 min to remove the surface nickel oxide layer of the NF and then rinsed with Millipore water (residual conductivity 18.2 MΩ·cm).

### 3.2. Synthesis of Modified Ketjenblack Carbon (mKB)

The amount of 10 g KB was dispersed into 1 L of HNO_3_ (20 wt.%) to form a homogeneous black dispersion that was heated to 80 °C under continuous stirring. After 3 h, the product was separated by centrifugation (10,000 rpm, 10 min) and washed with deionized water (500 mL) until the pH value was 7. Yield: 9.58 mg. This modified Ketjenblack carbon was denoted as mKB. Elemental analysis for Ketjenblack carbon: C 97.91 (O 2.91)% and modified Ketjenblack carbon: C 87.93, H 0.79 (O 11.28)%. IR (ATR, cm^−1^); 3435 (νOH), 1740 (νC=O), 1550 (ν_δ_OCO^−^), and 1181 (ν_s_C–OO).

### 3.3. Synthesis of NiDMOF

NiDMOF was synthesized by a solvothermal reaction according to the literature with a slight modification [75]. Ni(NO_3_)_2_·6H_2_O (183.12 mg, 0.63 mmol), H_2_BDC (104.66 mg, 0.63 mmol), and DABCO (36.33 mg, 0.32 mmol) were added into 16 mL of *N*,*N*-dimethylformamide (DMF). The resulting slurry was stirred overnight at room temperature to obtain a homogeneous dispersion. The clear solution was collected and transferred into a Pyrex tube and then heated at 120 °C for 48 h. After cooling to room temperature, the green crystalline powder was separated by centrifugation (10,000 rpm, 10 min) and washed three times with DMF (20 mL each). The washing process was repeated three more times with MeOH (20 mL each) and the product collected by centrifugation (10,000 rpm, 10 min). The product was activated at 120 °C under vacuum (<10^−2^ mbar) for at least 12 h and finally stored under nitrogen.

Yield: 200 mg (57% based on H_2_BDC). Elemental analysis for [Ni_2_(C_8_H_4_O_4_)_2_(C_6_H_12_N_2_)]·(H_2_O)_0.5_ (MW 566.79 g mol^−1^), calculated C 46.62, H 3.73, N 4.94, Ni 20.71 %; [Ni_2_(C_8_H_4_O_4_)_2_(C_6_H_12_N_2_)]·H_2_O (MW 575.81 g mol^−1^), calculated C 45.89, H 3.85, N 4.87, Ni 20.39%; found C 45.26, H 3.61, N 4.94, Ni 20.82%.

### 3.4. Synthesis of Ni(Fe)DMOF

The bimetallic Ni(Fe)DMOF was synthesized by a similar procedure to the monometallic NiDMOF. The amount of Ni(NO_3_)_2_·6H_2_O (177.65 mg, 0.61 mmol), H_2_BDC (104.66 mg, 0.63 mmol), and DABCO (36.33 mg, 0.32 mmol) were dispersed into 11 mL of DMF. The resulting slurry was stirred overnight at room temperature to obtain a homogeneous dispersion. To this solution, 0.02 mmol of Fe(OAc)_2_ (3.32 mg) in 5 mL of DMF was added dropwise (for an intended molar Ni:Fe ratio of 32:1). After sonicating for 30 min, the mixture was sealed into a Pyrex tube and heated at 120 °C for 48 h. After cooling to room temperature, the palm-leaf crystalline powder was separated by centrifugation (10,000 rpm, 10 min) and washed three times with DMF (20 mL each). The washing process was repeated three more times with MeOH (20 mL each) and the product collected by centrifugation (10,000 rpm, 10 min). The product was activated at 120 °C under vacuum (<10^−2^ mbar) for at least 12 h and stored under nitrogen.

Yield: 191 mg (54% based on H_2_BDC). Elemental analysis for [Ni_1.94_Fe_0.06_(C_8_H_4_O_4_)_2_(C_6_H_12_N_2_)] (H_2_O)_0.5_ (MW 566.61 g mol^−1^), calculated: C 46.63, H 3.74, N 4.94, Ni 20.10, Fe 0.59%; [Ni_1.94_Fe_0.06_(C_8_H_4_O_4_)_2_(C_6_H_12_N_2_)]·(H_2_O) (MW 573.88 g mol^−1^), calculated: C 45.90, H 3.85, N 4.87, Ni 19.78, Fe 0.58%; found C 46.35, H 3.57, N 5.01, Ni 20.85, Fe 0.66%.

### 3.5. Synthesis of NiDMOF/mKBx and Ni(Fe)DMOF/mKB14

The composites NiDMOF/mKBx and Ni(Fe)DMOF/mKB14 were synthesized according to the above procedures described for NiDMOF and Ni(Fe)DMOF, except that the modified Ketjenblack carbon (mKB) powder (12, 24, 40 and 66 mg) was dispersed in 3 mL of DMF by sonication for 30 min in advance and then added into the metal salt/H_2_BDC/DABCO/DMF solution. In order to determine the MOF and mKB content in the final composite, the metal wt.% in the NiDMOF/mKBx composites was determined by flame atomic absorption spectroscopy (AAS). From these metal wt.% of the AAS results, the mass fractions of the MOF were calculated from which the mKB wt.% in the composites was taken as the difference to 100% (Table S2, Supplementary Materials).

The NiDMOF/mKBx composites had mKB contents of x = 7, 14, 22, and 34 wt.%. For Ni(Fe)DMOF, the mKB content in the composite was 14 wt.%. NiDMOF is a green crystalline powder, but its mKB composites had an increasing darker color with increasing mKB content.

Yields: 61% NiDMOF/mKB7, 65% NiDMOF/mKB14, 70% NiDMOF/mKB22, 76% NiDMOF/mKB34, 62% Ni(Fe)DMOF/mKB14, based on H_2_BDC.

### 3.6. Materials Characterization

Powder X-ray diffraction (PXRD) data were collected with a Rigaku Miniflex 600 powder diffractometer (Rigaku, Tokyo, Japan) using a low background silicon sample holder and Cu-Kα irradiation (λ = 1.54184 Å). The measurements were conducted over a 2θ = 2–100° range with a scan speed of 1.5 deg min^−1^ (600 W, 40 kV, 15 mA). The diffractograms were analyzed using the software Match 3.1.0.

The Fourier transform infrared (FTIR) spectra of all the samples were recorded on a Bruker FTIR Tensor 37 spectrometer (Bruker AXS, Karlsruhe, Germany) in attenuated total reflection (ATR) mode with a diamond crystal or as KBr pellets in the range of 400–4000 cm^−1^.

^1^H MNR measurements were performed with a Bruker Advance III-300 (Bruker, Karlsruhe, Germany) operating at 300 MHz. Before dissolution under digestion for the solution NMR measurement, the MOF samples were activated at 150 °C for at least 20 h under vacuum (<10^−2^ mbar) in order to remove the residual solvent molecules. Then, the amount of 5 mg of each activated sample was dissolved with decomposition in 0.8 mL DMSO-d_6_ and 50 μL D_2_SO_4_.

Nitrogen sorption isotherms were obtained with a Belsorp MAXII (Microtrac MRB, Haan, Germany) high precision gas/vapor adsorption measurement instrument at 77 K and evaluated with the BELMaster MAXII software (version 7.3.2.0). Prior to the measurement, the materials were activated by degassing under vacuum (10^−2^ mbar) at 120 °C for 12 h. Brunauer–Emmett–Teller (BET) surface areas were determined from the nitrogen adsorption isotherms and the pore size distributions were derived by non-local density functional theory (NLDFT) calculations based on the “nitrogen at 77 K with slit pores” method. The total pore volumes were calculated from the adsorbed volume at p/p_0_ = 0.95.

Elemental analysis (CHNS) was carried out using an Elementar Analysensysteme vario MICRO cube instrument (Elementar Analysensysteme, Langenselbold, Germany). The samples were dried at 150 °C under a vacuum (<10^−2^ mbar) for at least 20 h prior before the measurement.

Flame atomic absorption spectroscopy (AAS) was conducted with a PinAAcle 900T from PerkinElmer (Perkin Elmer LAS GmbH, Rodgau-Jügesheim, Germany). Exactly weighted samples (15–20 mg) were heated and stirred with concentrated hydrochloric acid overnight. The solution was carefully filtered and diluted with Millipore water to a volume of 25 mL and again by a factor of 1:50 for the AAS measurements.

Scanning electron microscopy (SEM) images were collected with a Jeol JSM-65 10 LV QSEM (Jeol, Akishima, Japan) advanced electron microscope with a LaB_6_ cathode at 20 kV equipped with a Bruker Xflash 410 (Bruker AXS, Karlsruhe, Germany) silicon drift detector for energy-dispersive X-ray spectrometric (EDX) elemental composition analysis. The signals of Cu, Zn, and Au in the EDX spectra originated from the sample holder and from the sputtering of the sample with gold prior to the investigation.

X-ray photoelectron spectroscopy (XPS) measurements were made using an ULVAC-PHI VersaProbe II microfocus X-ray photoelectron spectrometer (ULVAC-PHI, Chigasaki, Japan) equipped with a polychromatic aluminum Kα X-ray source (1486.8 eV). Experimental XP spectra were fitted by the CasaXPS, version 2.3.19PR1.0 from Casa Software Ltd. Program (Casa Software Ltd., Teignmouth, UK). A standard Shirley baseline with no offset was used for the background correction. Binding energies were calibrated to the carbon 1s orbital with a binding energy of 284.8 eV. In the case of the Fe 2p spectra, an additional correction was necessary due to the presence of a Ni-LMM Auger peak.

### 3.7. Electrochemical Measurements

Electrocatalytic measurements were carried out on an Interface 1010E potentiostat from Gamry Instruments with an RRDE-3A station from ALS Japan using a three-electrode configuration. The system consists of a coiled platinum wire as a counter electrode and a reversible hydrogen electrode (RHE) from Gaskatel (Kassel, Deutschland) as a reference electrode. Working electrodes were fabricated by depositing the prepared materials on a rotating disc electrode (RDE, 5 mm diameter, 0.196 cm^2^ area), here, a rotating disc glassy carbon electrode (GCE, GC-RDE). The amount of 2.5 mg of MOF or MOF/mKB was dispersed in methanol (0.5 mL) with 25 µL of Nafion (around 5% in a mixture of water and lower aliphatic alcohols) as the binding agent. The suspension was sonicated for 30 min to form a homogeneous ink. Then, 10 µL of the prepared ink was dropped onto the rotating disc electrode (loading 0.25 mg cm^−2^) and dried at room temperature. As an electrolyte, 1 mol L^−1^ KOH solution (pH: 14) was used and nitrogen was purged for 10 min through the cell to remove oxygen before the test. The working electrode was kept rotating at a rate of 1600 rpm during the measurements. The electrode was pre-cycled for at least 20 cycles at a sweep rate of 100 mV cm^–1^ between 1.0 and 1.7 V until reaching the steady state. All linear scanning voltammetry (LSV) curves were collected by sweeping the potential from 0.8 to 1.7 V vs RHE with a scan rate of 5 mV s^−1^. Cyclic voltammetry (CV) measurements were carried out in the potential range between 1.0 and 1.7 V vs. RHE with a scan rate of 100 mV s^−1^. iR compensation was completely applied to the recorded data afterward, as recommended in the literature [122]. In all measurements, R_u_ (uncompensated electrolyte resistance) was measured by electrochemical impedance spectroscopy (EIS) at the open circuit potential (OCP) and the iR_u_ drop was compensated automatically by the R_u_ value using the software Gamry Echem Analyst., Version 7.8.2.

The overpotential η was derived from the standard potential E vs. RHE using the formula:

η = ERHE − 1.23 V.(6)

The Tafel slope b was obtained by fitting the linear portion of the Tafel plots, which were derived from the LSV curves according to the Tafel equation:

η = a + b × log(j).(7)

where a is a constant and j represents the current density. Electrochemical impedance spectroscopy (EIS) data were recorded with the frequency range of 0.1–10,000 Hz at the potential of 1.5 V. To check the long-term stability of the best catalyst material at a constant current by chronopotentiometry, a Ni(Fe)DMOF/mKB@NF electrode was fabricated by drop-casting the catalyst ink on the surface of pre-treated nickel foam and drying at room temperature. The loading was around 1 mg cm^−2^ by weighing the electrode before and after material deposition. The chronopotentiometry at 50 mA cm^−2^ was carried out with 1 cm^2^ of commercial nickel foam (NF) for 30 h in 1 mol L^−1^ KOH electrolyte.

## 4. Conclusions

In summary, we successfully fabricated a series of novel DMOF/mKB composites containing a bimetallic nickel-iron-based pillared MOF (DMOF) and modified Ketjenblack carbon using a simple one-step hydrothermal procedure, which can be directly used as efficient electrocatalysts in OER. The appropriate mass weight % of mKB in the composite DMOF/mKB is important for the fabrication of the optimum catalyst. Benefitting from enhanced conductivity, remarkably high surface areas, and the high intrinsic catalytic activity of mKB and the integration of Ni and Fe active sites from the MOF precursors, the Ni(Fe)DMOF/mKB14 with 14 wt.% mKB exhibited superior OER performance, which only needed a low overpotential of 279 mV and a Tafel slope of 25 mV dec^−1^ to reach the current density of 10 mA cm^−2^ in 1 mol L^−1^ of KOH electrolyte, outperforming the benchmark RuO_2_ catalyst and many state-of-the art MOF-based and Ni-Fe-based OER catalysts. For the practical application, the working electrode was fabricated by depositing optimized Ni(Fe)DMOF/mKB14 electrocatalysts on commercial nickel foam, where the current density at 10 and 50 mA cm^−2^ was realized at 247 and 291 mV, respectively, and the activity was maintained for 30 h of applied bias. The superior OER performance is associated with the transformation of Ni(Fe)DMOF into highly functionalized α/β-Ni(OH)_2_/β/γ-NiOOH and FeOOH inherited from the DMOF structure. The presented DMOF/mKB material preparation strategy is applicable to the development of high-performance electrode materials for other advanced energy storage systems.

## Figures and Tables

**Table 1:** The BET surface area and total pore volume of the NiDMOF and DMOF/mKBx composites.

Material	BET Surface Area ^a^ (Estimated) ^c^ (m^2^ g^−1^)	Total Pore Volume ^b^ (Estimated) ^c^ (cm^3^ g^−1^)
NiDMOF-Literature [73]	2050	0.80
NiDMOF	2104	0.82
NiDMOF/mKB7	1897 (2043)	0.86 (0.87)
NiDMOF/mKB14	1773 (1982)	1.18 (0.92)
NiDMOF/mKB22	1106 (1913)	0.70 (0.97)
NiDMOF/mKB34	1087 (1808)	0.85 (1.05)
Ni(Fe)DMOF	1942	0.80
Ni(Fe)DMOF/mKB14	1486 (1843)	0.86 (0.90)
KB	1300	1.76
mKB	1234	1.50

^a^ BET surface area calculated at 0.002 < p/p_0_ < 0.15 from the N_2_ sorption isotherm 77 K with a standard deviation ± 20 m^2^∙g^−1^. ^b^ The total pore volumes were determined at p/p_0_ = 0.95 from the adsorption branch for pores ≤ 25 nm. ^c^ Estimated BET surface area as the sum of the mass-weighted surface areas of NiDMOF (2104 m^2^∙g^−1^) and mKB (1234 m^2^∙g^−1^) calculated from the following formula: (1) $\mathrm{BET}\;\left(\mathrm{estimated}\right)=\frac{\mathrm{wt}.\%\;\mathrm{NiDMOF}}{100}\times 2104{\;m}^{2}\cdot g^{-1}+\frac{\mathrm{wt}.\%\;\mathrm{mKB}\;}{100}\times 1234{\;m}^{2}\cdot g^{-1}$
The estimated total pore volume was calculated accordingly. NiDMOF/mKB7 stands for 93 wt.% NiDMOF and 7 wt.% mKB.

## Data Availability

The data presented in this study are available on request from the corresponding author.

## Supplementary Materials

The following supporting information can be downloaded at: https://www.mdpi.com/article/10.3390/molecules28114464/s1, Section S1: 3D framework structure of NiDMOF; Section S2: Powder X-ray diffraction (PXRD) measurements; Section S3: Fourier-transform infrared (FT-IR) spectroscopy; Section S4: NMR spectroscopy; Section S5: Elemental analysis; Section S6: Nitrogen sorption experiments (T = 77 K); Section S7: Scanning electron microscopy (SEM); Section S8: X-ray photoelectron spectra (XPS); Section S9: Electrochemical measurements. References [123,124,125,126,127,128,129,130,131] are cited in the Supplementary Materials.

## Sample Availability

Samples of the compounds will not be available.
