# Supplementary material for: Synthesis of Ketjenblack Decorated Pillared Ni(Fe) Metal-Organic Frameworks as Precursor Electrocatalysts for Enhancing the Oxygen Evolution Reaction
Source: Molecules. 2023 May 31;28(11):4464. doi: 10.3390/molecules28114464 (PMC10254712; doi:10.3390/molecules28114464)
Supplement: Supplementary file 1 [file molecules-28-04464-s001.zip › molecules-2373905-supplementary.pdf]

Supplementary Materials

Synthesis of Ketjenblack Decorated Pillared Ni(Fe) Metal-Organic Frameworks as Precursor Electrocatalysts for Enhancing the Oxygen Evolution Reaction

Thi Hai Yen Beglau[1], Lars Rademacher[1], Robert Oestreich[1] and Christoph Janiak[*1,§]

Address:
[1]Institut für Anorganische Chemie und Strukturchemie, Heinrich-Heine-Universität Düsseldorf, 40204 Düsseldorf, Germany,
E-Mail:
Christoph Janiak* - janiak@uni-duesseldorf.de
* Corresponding author
§ Fax: +49-211-81-12287; Tel: +49-211-81-12286
Emails: beglau@uni-duesseldorf.de; lars.rademacher@hhu.de; Robert.Oestreich@hhu.de

Section S1: 3D framework structure of NiDMOF

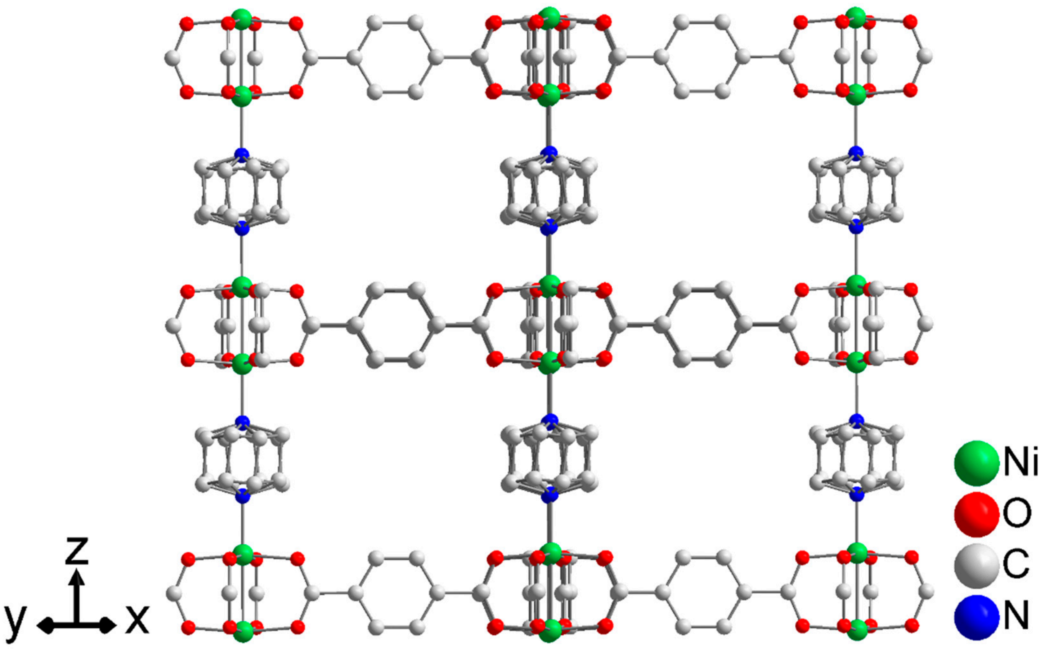

(a)

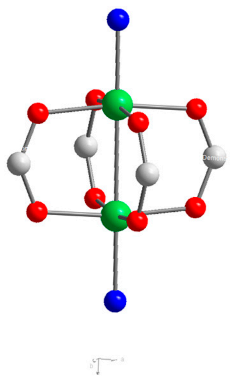

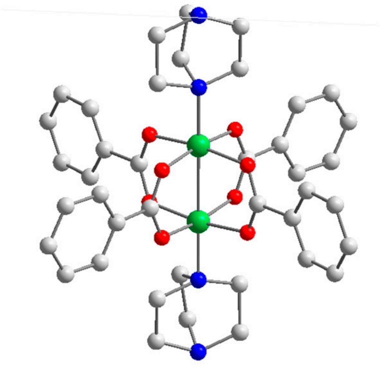

(b) **(c)**

Figure S1. a) Secion of the 3D framework structure of $[Ni_2(BDC)_2DABCO]\cdot(DMF)_4\cdot(H_2O)_{1.5}$ (NiDMOF), b) and c) two presentations of the $\{Ni_2(O_2C\text{-})_4\}$ paddle-wheel cluster also showing the penta-coordination of the Ni centers by the nitrogen atoms from the DABCO ligand. Graphic produced with the software Diamond [1] from the deposited cif-file for NiDMOF with CCDC number 802892.[2]

Section S2: Powder X-ray diffraction (PXRD) measurements

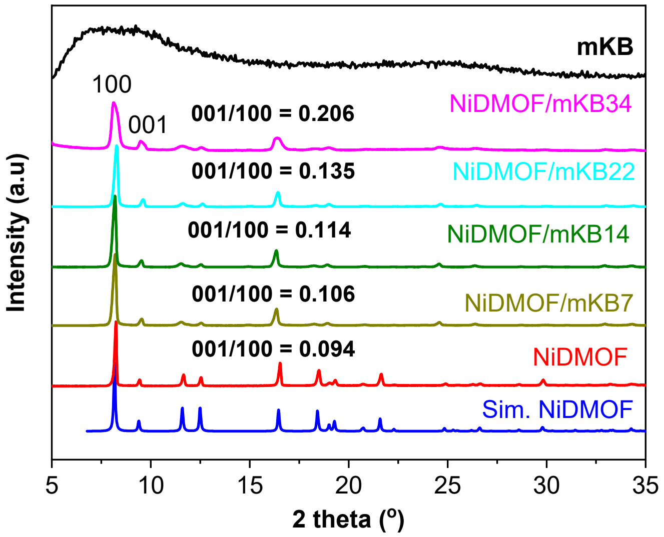

Figure S2. PXRD patterns of NiDMOF, its 7, 14, 22, 34 wt.% mKB composites and mKB. The simulated PXRD pattern of NiDMOF was obtained from the deposited cif-file for NiDMOF with CCDC number 802892.[2] The first five reflexes at 2θ = 8.2°, 9.4°, 11.7°, 12.4° and 16.6° correspond are the 100, 001, 110, 101 and 200 reflections.

Section S3: Fourier-transform infrared (FT-IR) spectroscopy

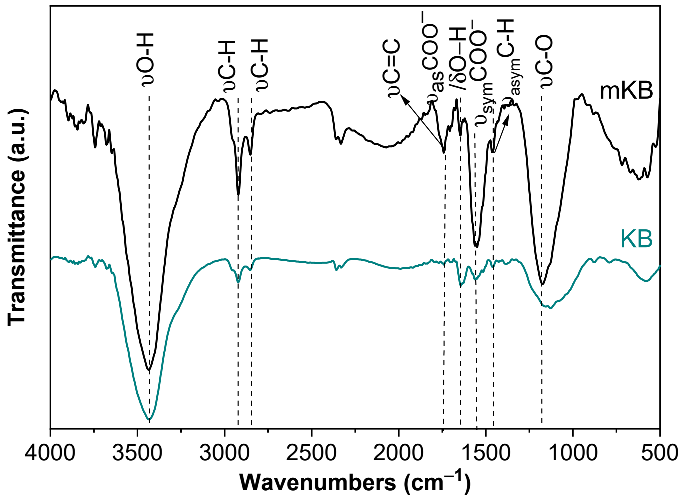

Figure S3. FTIR spectra of modified Ketjenblack carbon (mKB) and pristine Ketjenblack carbon (KB).

Fourier-transform infrared (FT-IR) spectroscopy was used to confirm the existence and increase of functional groups in mKB (Figure S3). The spectrum of mKB shows peaks at 3435, which could be attributed to the ν(O–H). The bands at 2921 and 2850 cm^{-1} are assigned to the ν_{asym}C–H and ν_{sym}C-H, respectively. After modification, the mKB has additional bands at 1640 ± 5, 1550 ± 5 and 1180 ± 5 cm^{-1},

attributed to the $\nu_{asym}COO^{-}$, $\delta O\text{–}H$, $\nu_{sym}COO^{-}$ and $\nu C\text{–}O$, respectively.[3] The oxygen containing groups –OH, -COO⁻ and -C-O can be obserbed in both KB and mKB, but the intensity in mKB is much stronger than in KB, indicating that oxidation with 20% HNO_3 increased the amount of oxygen containing functional groups.

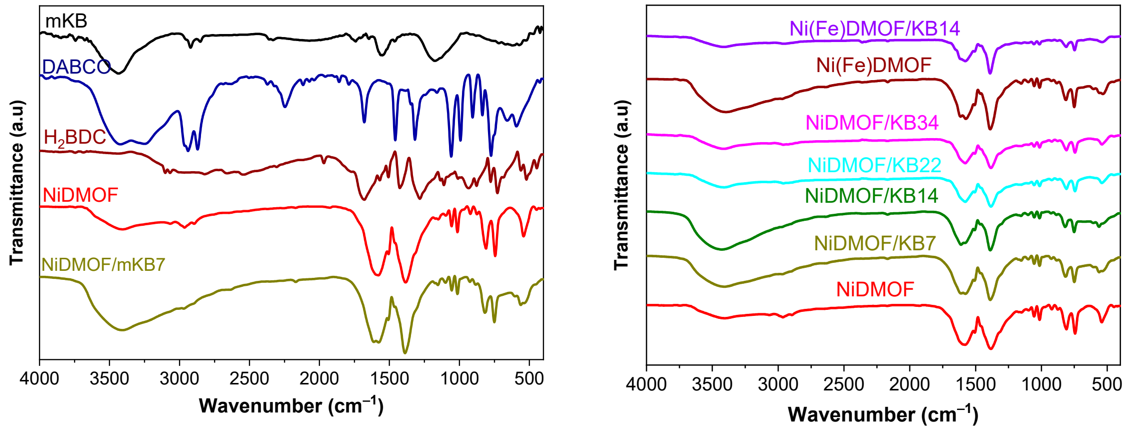

(a) **(b)**

Figure S4. FT–IR spectra of a) mKB, H_2BDC, DABCO, NiDMOF and the Ni-DMOF/KB7 composite; b) NiDMOF and its 7, 14, 22, 34 wt.% mKB composites, Ni(Fe)DMOF and Ni(Fe)DMOF/mKB14.

As shown in Figure S4a, NiDMOF and NiDMOF/KB7 display the same characteristic bands in the fingerprint region which are in a good agreement with the literature.[2,4] The wide band at 2900–3400 cm^{-1} are attributed to $\nu_{asym}C\text{–}H$ and $\nu O\text{–}H$ of uncoordinated water molecules. The strong bands at 1650-1580 cm^{-1} and 1400-1350 cm^{-1} were assigned to the $\nu_{asym}COO^{-}$ and $\nu_{sym}COO^{-}$ vibrations of the COO^{-} groups. The bands at 1100 ± 5 cm^{-1} and 1055 ± 5 cm^{-1} could be due to the $\nu_{as}C\text{-}N$ and $\nu_{as}C\text{-}N$ vibrations of DABCO. The absorbance bands at 810 ± 5 cm^{-1} and 750 ± 5 cm^{-1} correspond to the $\nu(C\text{-}C)_{Ar}$ and $\delta(C\text{-}H)_{Ar}$ vibrations, respectively, of the 1,4-BDC ligand. The composite series DMOF/mKBx exhibit similar bands as those in the parent DMOF, indicating that incorporation of mKB did not prevent the formation of DMOF units. The results confirmed that mKB could be smoothly mixed with the DMOFs, in agreement with the PXRD results.

Table S1. Assignment of IR-bands of KB, mKB, NiDMOF and NiDMOF/mKB analogs (cm^{-1}).

	νO–H	νC-H	$\nu_{asym}COO^{-}/\delta$O-H	$\nu_{sym}COO^{-}$	$\nu_{s}CO^{-}$	ν_{as}C-N	νC-C_{Ar} / δ(C-H)$_{Ar}$	νNi-O/ νNi/Fe–O,
**KB**	3435	2918<br>2844	1641<br>1557	-	1174	-	879<br>722	-
**mKB**	3435	2918<br>2844	1645<br>1554	-	1181	-	875<br>782<br>722	-
**NiDMOF**	3406	2963<br>2887	1602<br>1573	1385	1147	1056<br>1010	813<br>737	540
**NiDMOF/mKB**	3430	2961<br>2895	1611<br>1573	1387	1144	1048<br>1005	817<br>715	560
**Ni(Fe)DMOF**	3408	2970	1615<br>1568	1390	1150	1055<br>1007	748<br>814	528
**Ni(Fe)DMOF/mKB**	3401	2918<br>2849	1618<br>1572	1390	1160	1053<br>1007	748<br>814	527
**Derived- Ni(Fe)DMOF[b]**	3637<br>3425	-	1633	1347	-	-	-	517<br>439
**Derived- Ni(Fe)DMOF/mKB[b]**	3634<br>3430	-	1641<br>1570	1367	-		-	516<br>450

[a] ν = stretching vibration (ν_{asym}= asymmetric vibration, ν_{sym}=symmetric stretching vibration); δ =bending vibration (ρ = in plane, γ= out of plane vibration); $_{Ar}$ = aromatic vibration.

[b] All major IR bands are listed for the decomposition product of Ni(Fe)DMOF and Ni(Fe)DMOF/mKB after 24 h in 1 mol L^{-1} KOH.

Section S4: NMR spectroscopy

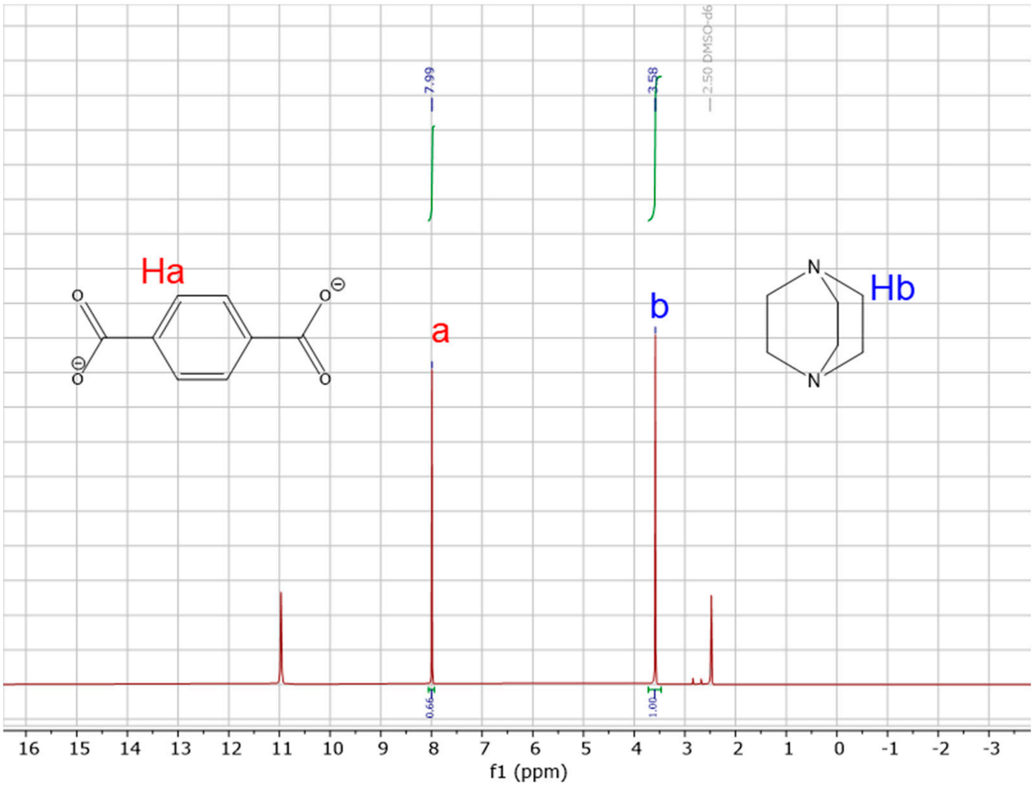

Figure S5. Solution ^{1}H NMR spectra of digested NiDMOF [$Ni_2(BDC)_2DABCO$] in DMSO-d_6 (2.50 ppm). The intergration ratio of H on 1 DABCO ($C_6H_{12}N_2$) to H on 2 BDC (2 $C_6H_4(CO_2^-)_2$) matches the expected 1.5:1 ratio.

Section S5: Elemental analysis

Table S2. Elemental analysis of DMOF samples and their composite series with mKB.[a]

Material	C (wt.%)	H (wt.%)	N (wt.%)	Ni (wt.%)	Fe (wt.%)	mKB from AAS (wt.%)
NiDMOF found	45.26	3.61	4.94	20.82	-	-
NiDMOF = $[Ni_2(C_8H_4O_4)_2(C_6H_{12}N_2)]\cdot(H_2O)_{0.5}$ calculated	46.62	3.73	4.94	20.71	-	-
$[Ni_2(C_8H_4O_4)_2(C_6H_{12}N_2)]\cdot H_2O$ calculated	45.89	3.85	4.87	20.39		
NiDMOF/mKB7	46.28	3.36	4.24	19.49	-	7
NiDMOF/mKB14	48.84	3.19	4.18	18.13	-	14
NiDMOF/mKB22	50.20	3.11	3.88	16.44	-	22
NiDMOF/mKB34	51.86	3.00	3.69	13.98	-	34
Ni(Fe)DMOF	46.35	3.57	5.01	20.85<br>3.55 at.%	0.66<br>0.118 at.%	-
			molar ratio Ni:Fe 30:1			
Ni(Fe)DMOF= $[Ni_{1.94}Fe_{0.06}(C_8H_4O_4)_2(C_6H_{12}N_2)]\cdot(H_2O)_{0.5}$ calc.	46.63	3.74	4.94	20.10	0.59	-
$[Ni_{1.94}Fe_{0.06}(C_8H_4O_4)_2(C_6H_{12}N_2)]\cdot(H_2O)$ calc.	45.90	3.85	4.87	19.78	0.58	
Ni(Fe)DMOF/mKB14	49.17	3.28	4.27	18.11<br>3.086 at.%	0.57<br>0.102 at.%	14
			molar ratio Ni:Fe 30:1			

[a] The samples were dried at 150 °C for at least 20 h under vacuum condition ($< 10^{-2}$ mbar) before using for the element analysis, in order to avoid the residual solvent molecules or re-adsorbed moisture during storage and handling.

[b] $\text{MOF wt.\% in composite} = \frac{\text{Ni wt.\% (composite)}}{20.71\ x\ \text{Ni wt.\% (pure MOF)}} \cdot 100\%$; mKB wt.% = 100% – MOF wt.%

Section S6: Nitrogen sorption experiments (T = 77 K)

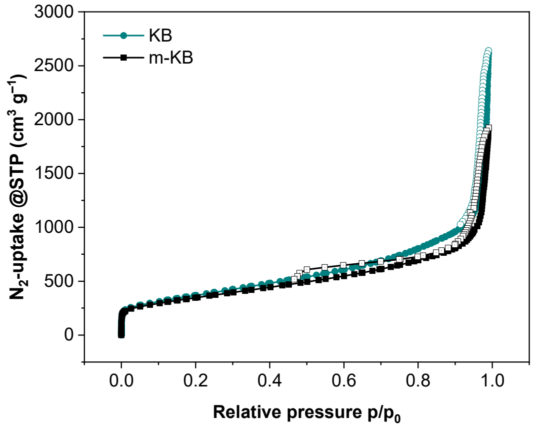

(a)

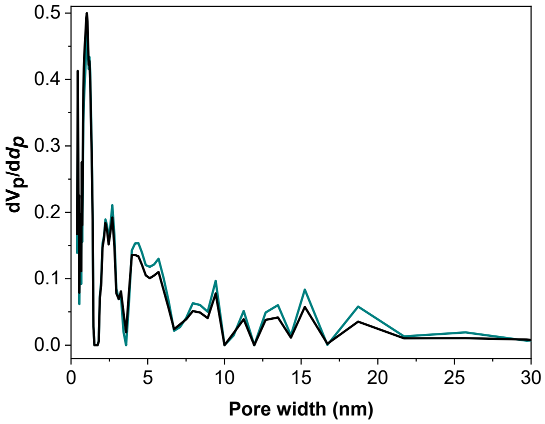

(b)

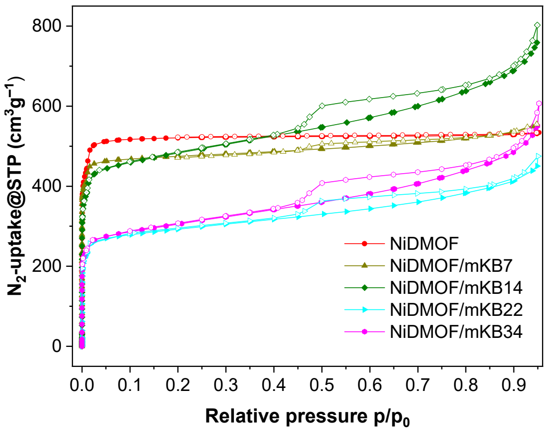

(c)

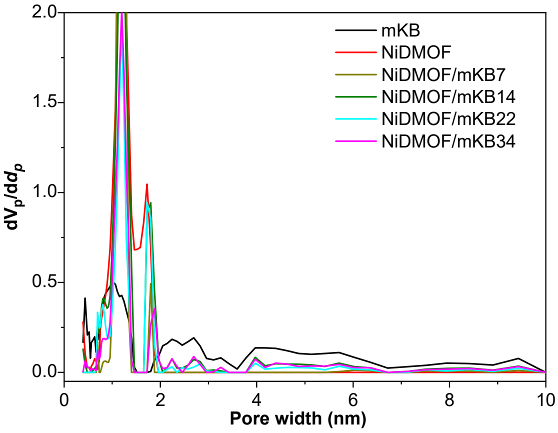

(d)

Figure S6. a) Nitrogen sorption isotherms at 77 K (filled symbols adsorption; empty symbols desorption) and b) pore size distribution of modified Ketjenblack carbon (mKB) and neat Ketjenblack carbon (KB). c) Nitrogen sorption isotherms at 77 K (filled symbols adsorption; empty symbols desorption) and d) pore size distribution of NiDMOF, its 7, 14, 22, 34 wt.% mKB composites and mKB.

Section S7: Scanning electron microscopy (SEM)

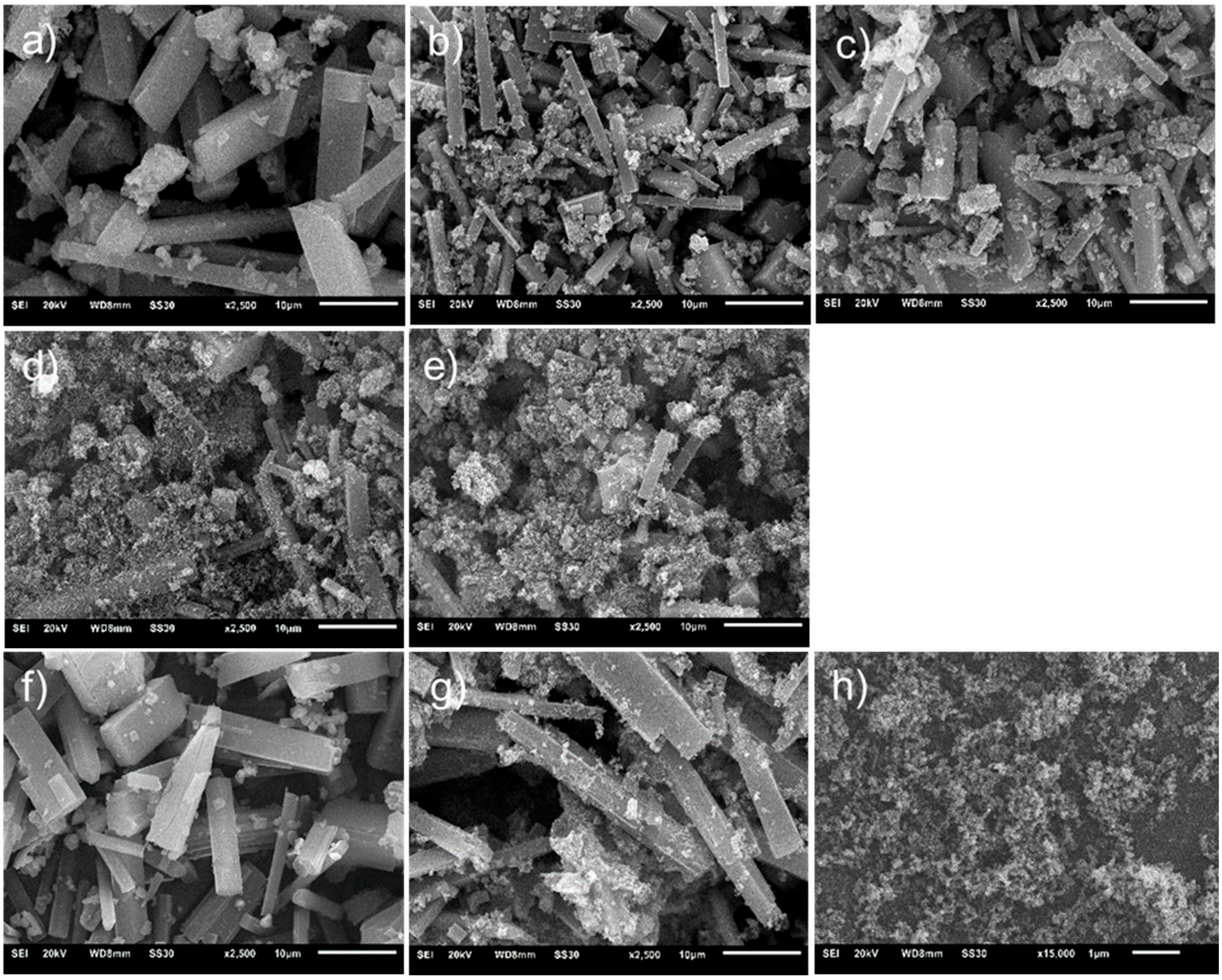

Figure S7. SEM images of a) NiDMOF, b) NiDMOF/mKB7 c) NiDMOF/mKB14 d) NiDMOF/mKB22, e) NiDMOF/mKB34, f) Ni(Fe)DMOF, g) Ni(Fe)DMOF/mKB14 and h) mKB. The scale bar is 10 μm except for h) where it is 1 μm.

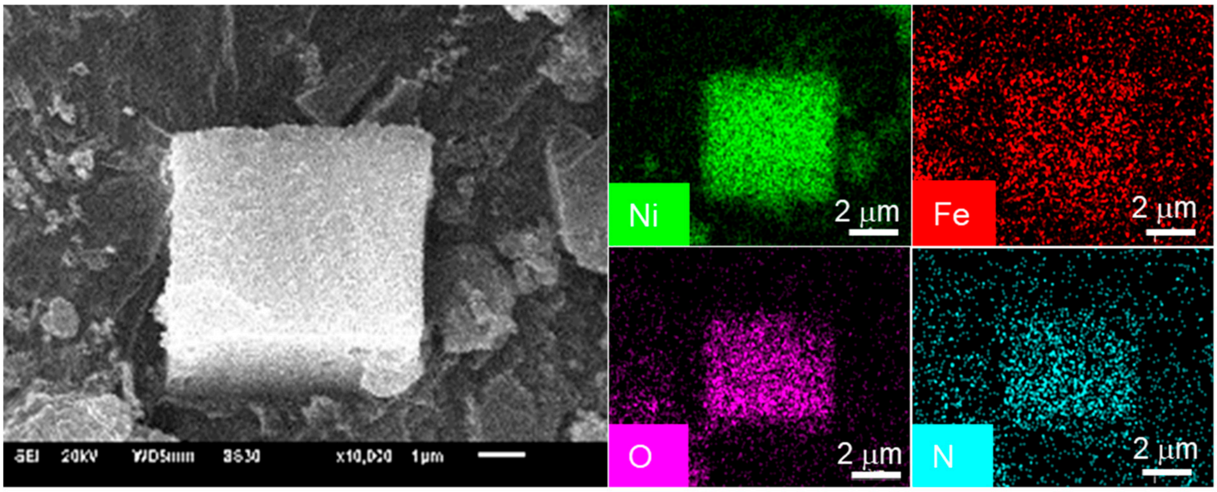

Figure S8. EDX element mapping for nickel, iron, nitrogen and oxygen of Ni(Fe)DMOF.

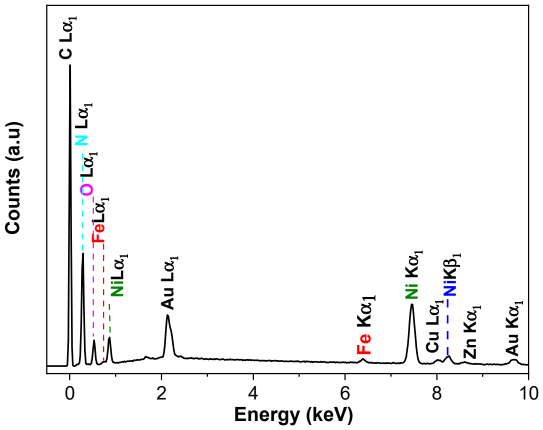

(a)

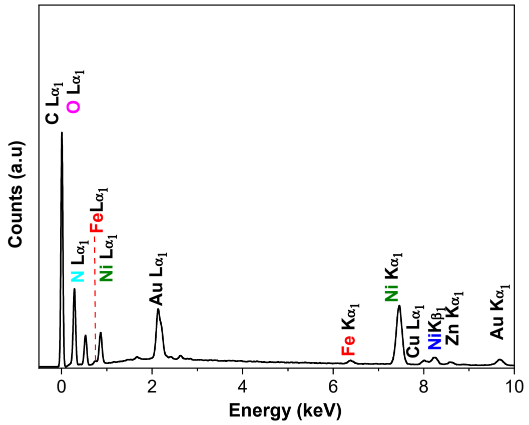

(b)

Figure S9. EDX spectra with the composition for a) Ni(Fe)DMOF in the selected area in Figure S8, b) for Ni(Fe)DMOF/mKB14 in the selected area in Figure 3 in the main text.

Table S3. SEM-EDX and AAS Ni:Fe ratios of Ni(Fe)DMOF and Ni(Fe)DMOF/mKB14.

**Material**	**SEM-EDX**	**AAS**	**Synthesis**
	**Molar Ni:Fe ratio**		
Ni(Fe)DMOF	31.5:1	30:1	32:1
Ni(Fe)DMOF/mKB14	31:1	30:1	32:1

Section S8: X-ray photoelectron spectra (XPS)

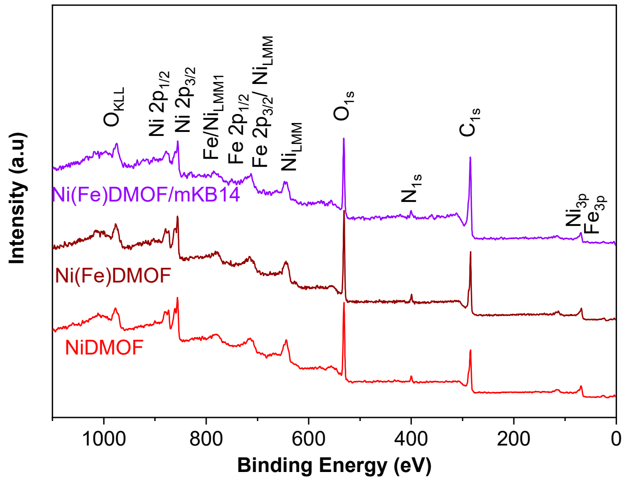

Figure S10. Full X-ray photoelectron spectra (XPS) of NiDMOF, Ni(Fe)DMOF and Ni(Fe)DMOF/mKB14.

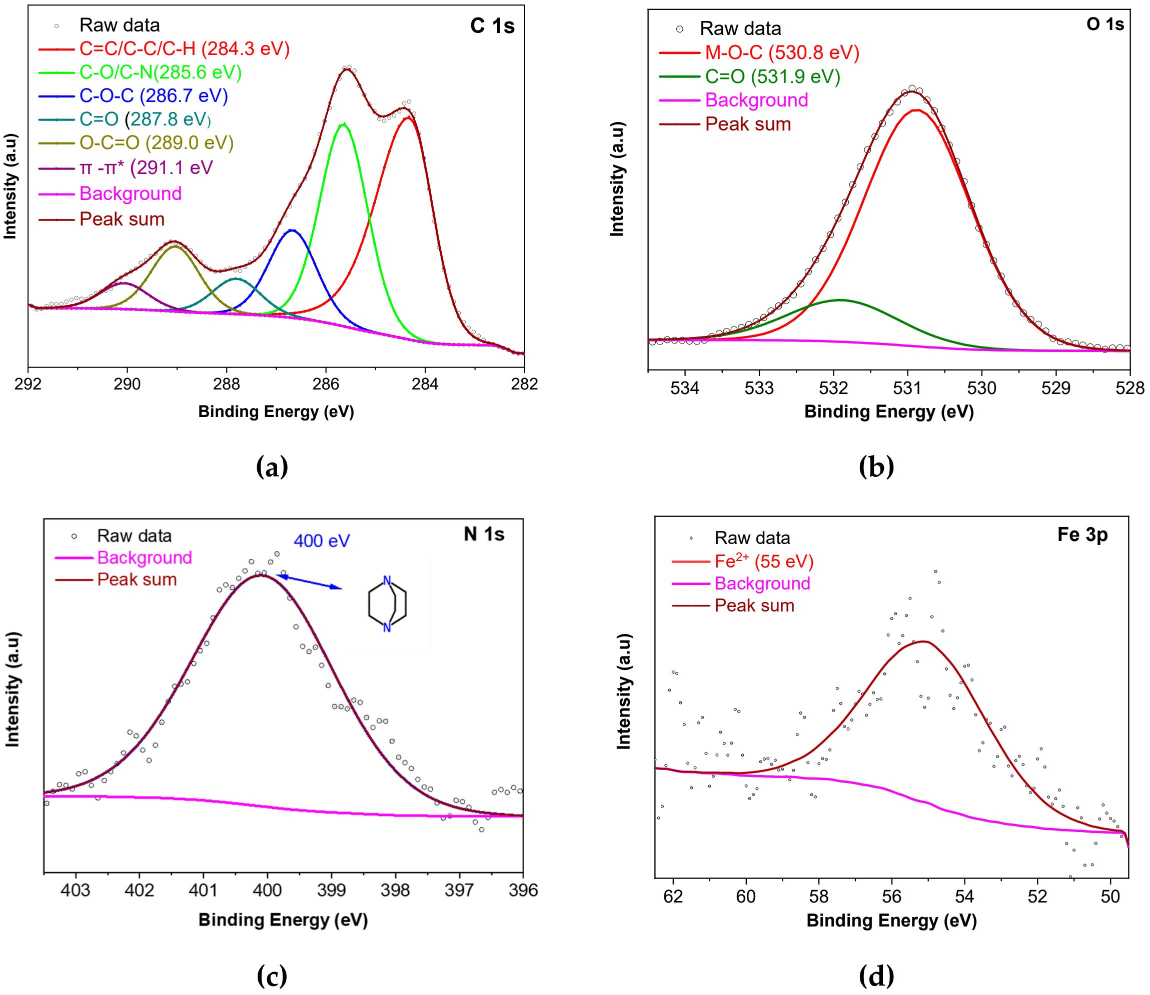

Figure S11. High-resolution X-ray photoelectron spectra (XPS) of a) C 1s, b) O 1s, c) N 1s and d) Fe 3p of Ni(Fe)DMOF/mKB14 composite.

The deconvolution of the carbon 1s spectrum (C 1s) can be fitted into five peaks with binding energies of 284.3, 285.6, 286.7, 287.8 and 289.0 eV, which indicate the presence of C-C/C=C/C-H, C-O/C-N, C-O-C, C=O and O-C=O, respectively. The shallow broad peak at 291.1 eV is a π to π* shake-up satellite.[5] This implies the presence of terephthalate and the functional groups of modified Ketjenblack carbon (mKB). Similarly, the oxygen spectrum (O 1s) can be deconvoluted into two peaks positioned at 530.8 and 531.9 eV, which are related to typical M-O-C bonds and C=O, respectively. The N 1s spectrum consists of one peaks with a binding energy of 400 eV which corresponds to the presence of tertiary N bonded to carbon ($N-C_3$),[6,7] as in DABCO. It should be noted that the Fe 2p peaks have a contribution from a Ni LMM Auger peak (Figure S11).[8] Therefore, the weaker Fe 3p peak was used, instead, for the Ni:Fe ratio quantification. The Fe 3p XPS region of Ni(Fe)DMOF/mKB shows one peak at 55 eV, which confirms of the presence of Fe(II) (Figure S11d).[9,10]

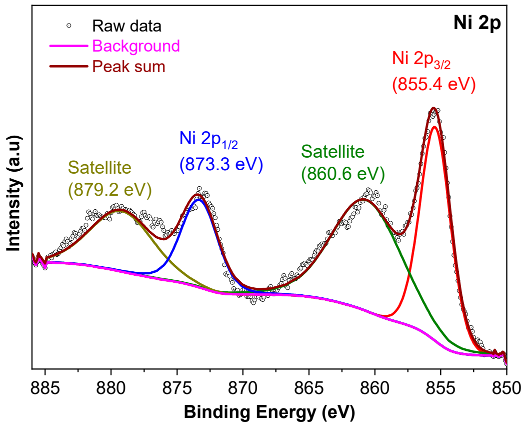

(a)

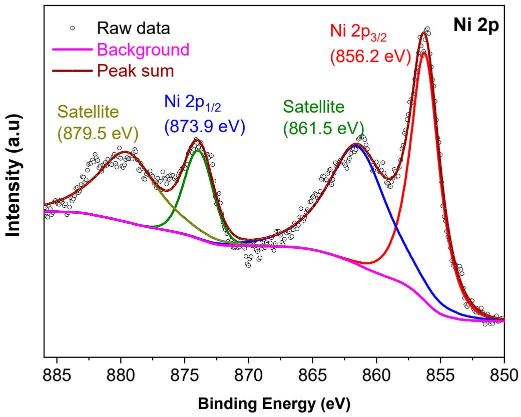

(b)

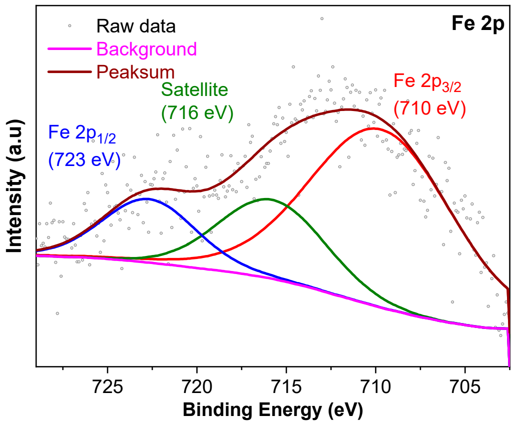

(c)

Figure S12. High-resolution X-ray photoelectron spectra (XPS) of a) Ni 2p of NiDMOF, b) Ni 2p and c) Fe 2p of Ni(Fe)DMOF. In c) the Ni LMM Auger peak occurs at the same positions as the Fe 2p signals.[8]

Section S9: Electrochemical measurements

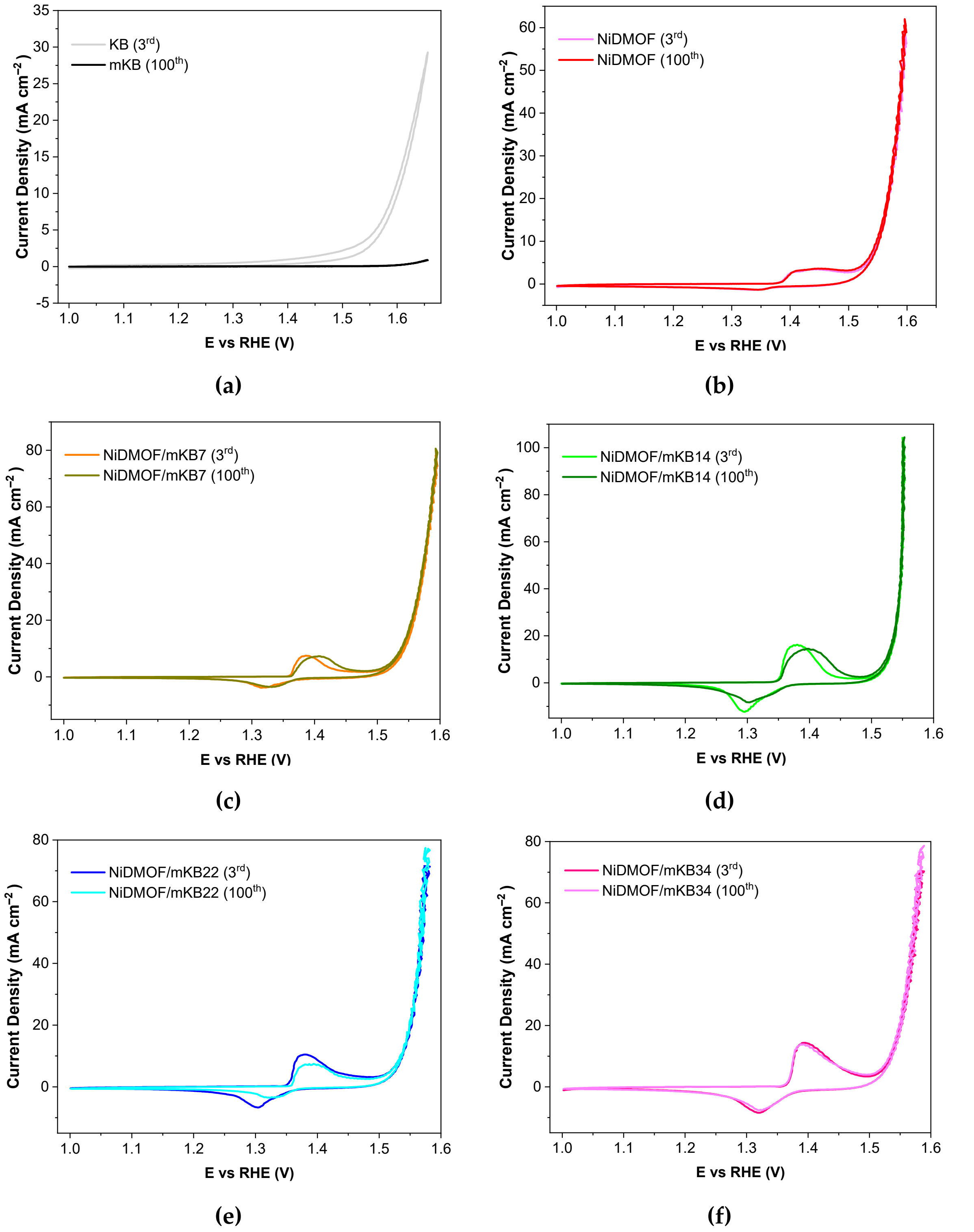

Figure S13. Comparison of CV curves collected after the 3rd and 100th cycle for a) mKB, b) NiDMOF, c) NiDMOF/mKB7, d) NiDMOF/mKB14, e) NiDMOF/mKB22 and f) NiDMOF/mKB34.

100 cyclic voltammetry (CV) scans were applied to explore the electrocatalytic behaviors and their activation/deactivation profiles due to surface reconstruction. As shown in Figure S13,

with mKB present in the composites, the Ni^{2+} to Ni^{3+} oxidation peak (at 1.35–1.45 V vs RHE) became more noticeable and shifted into positive direction, indicating the synergetic effect of mKB and NiDMOF on the oxidation of $Ni^{+2}(OH)_2$ to $Ni^{+3}OOH$. Oxidation currents at higher potentials are then due to the evolution of oxygen according to $4OH^- \rightarrow O_2 + 2H_2O + 4e^-$.

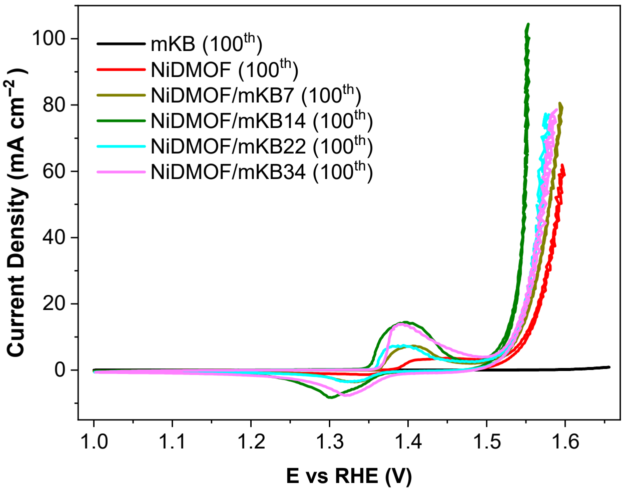

Figure S14. Comparison of CV curves collected after 100th cycle for mKB, NiDMOF and its 7, 14, 22, 34 wt.% mKB composites.

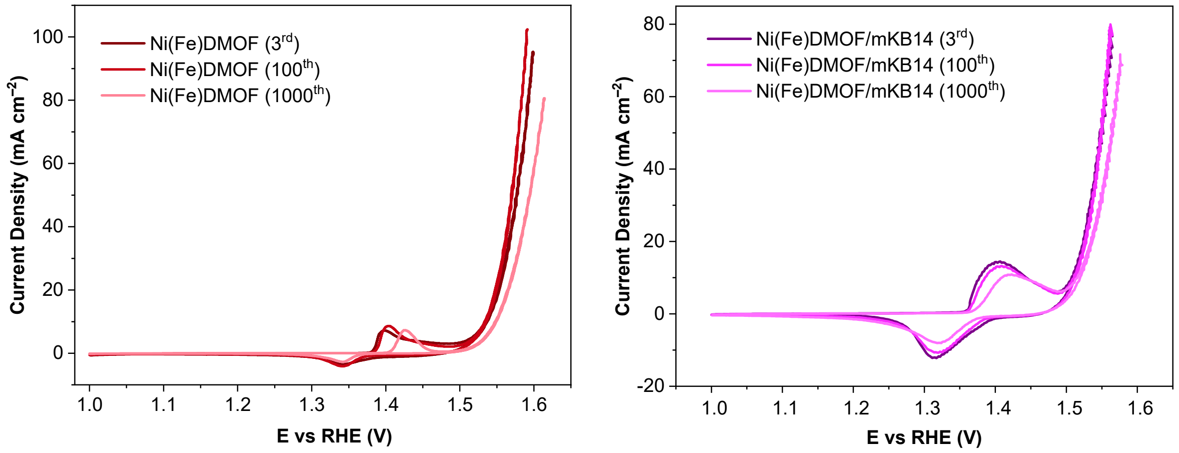

(a) (b)

Figure S15. Comparison of CV curves collected after 3rd, 100th and 1000th cycle for a) Ni(Fe)DMOF and b) Ni(Fe)DMOF/mKB14.

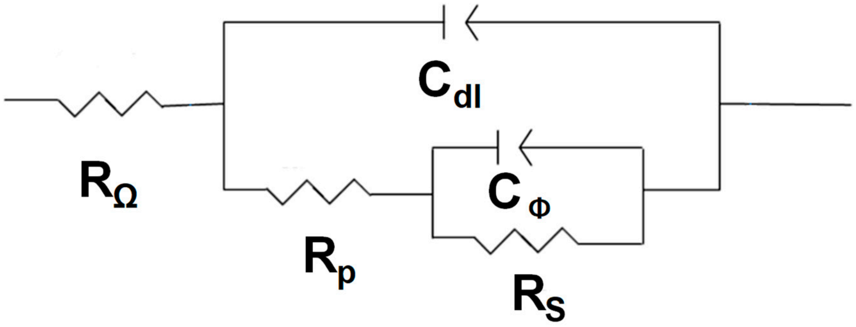

Figure S16. Equivalent circuit for the metal oxides catalyzing OER.

The R_{Ω} presents an electrolyte resistance, while C_{dl} is an element model, which represents the double layer capacitance of a solid electrode. The kinetic of Faradaic OER is determined by these circuit parameters: R_p, R_s and C_{Φ}. The polarization resistance (R_p) presents the total charge transfer resistance of the multiple steps in OER, while R_s is related to the rate of production of surface intermediates during OER. In general, C_{Φ} is related to the changes in charged surface species during the process of OER.[11,12,13]

Table S4. Overpotentials at 10 mA cm^{-2} and Tafel slopes of NiDMOF, Ni(Fe)DMOF and their mKB composite series materials at 1.5 V vs. RHE.

Material	Overpotential at 10 mA cm^{-2} (mV)	Tafel slope (mV dec^{-1})	R_p (Ω)	R_s (Ω)
Ketjenblack (mKB)	375	73	62	75
RuO_2	317	56	23	4
NiDMOF	315	55	21	7
NiDMOF/mKB7	308	53	8	3
NiDMOF/mKB14	294	32	4	2
NiDMOF/mKB22	303	45	5	3
NiDMOF/mKB34	304	51	11	4
Ni(Fe)DMOF	301	40	6	4
Ni(Fe)DMOF/mKB14	279	24	4	2

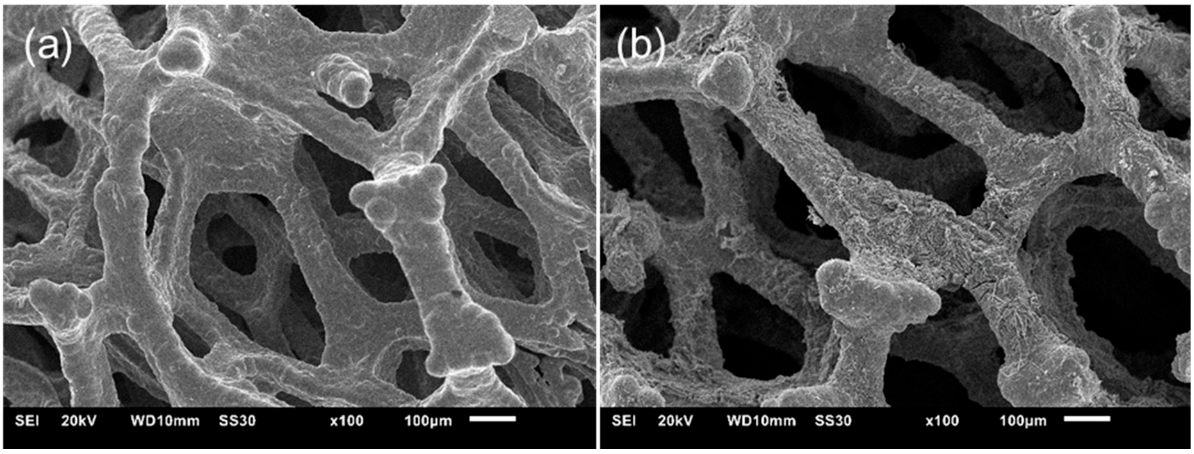

Figure S17. SEM images of (a) bare Ni foam (NF) and (b) Ni(Fe)DMOF/mKB14 loaded on nickel foam for the chronopotentiometry (CP) test for 30 h.

Table S5. Comparison of OER performance of published materials and in this work.

Material [a]	Electrolyte [b]	Substrate	Overpotential @ 10 mA cm^{-2} (mV)	Tafel slope (mV dec^{-1})	Ref.
NiDMOF/mKB14	KOH [b]	GCE	294	32	This work
Ni(Fe)DMOF/mKB14 (14 wt.% mKB)	KOH	GCE	279	25	This work
Ni(Fe)DMOF/mKB14 (14 wt.% mKB)	KOH	NF	247		This work
Ni(Fe)-MOF-74/KB (48 wt.% KB)	KOH	GCE	274	40	[14]
$Ni(Fe)(OH)_2$/KB (47 wt.%)	KOH	GCE	265	55	[14]
Ni10CoBTC/KB (33 wt.% KB)	KOH	GCE	347	70	[15]
Ni10FeBTC	KOH	GCE	344	47	[15]
2D-Ni-single-layer (Ni_2 BDC_2 DABCO)	PB, pH = 7.0 (0.2 mol L^{-1})	Carbon cloth	749	182	[16]
2D-CoNi-single-layer ($NiCoBDC_2DABCO$)	PB, pH = 7.0 (0.2 mol L^{-1})	Carbon cloth	527	171	[16]
HXP@NC800	KOH	GCE	307	48	17
Fe-doped HXP@NC800	KOH	GCE	266	49	[17]
$Ni_{32}Fe$ oxide	KOH	NF	291	58	[18]
β-$Ni(OH)_2$ nanoburls	KOH	GCE	303	43	[19]
α-$Ni(OH)_2$	KOH	GCE	387	53	[20]
α-$Ni(OH)_2$-GO	KOH	GCE	356	50	[20]

[a] mKB: modified Ketjenblack carbon, KB: Ketjenblack carbon; NF: nickel foam, BDC: 1-4-bezene dicarboxylate, TED = DABCO: triethylenediamine, HXP: hexagonal 2D $Ni_2(BDC)_2(DABCO)\cdot(DMF)_4(H_2O)_4$, rGO: reduced graphene oxide and PB: Sodium Phosphate Buffer 0.2 mol L^{-1} electrolyte, pH 7.0. – [b] 1 mol L^{-1} KOH electrolyte.

References

1 Brandenburg, K. Diamond, Version 4.6, Crystal and Molecular Structure Visualization, Crystal Impact; K. Brandenburg & H. Putz Gbr: Bonn, Germany, 2009–2022.

2 Maniam, P.; Stock, N. Investigation of Porous Ni-Based Metal–Organic Frameworks Containing Paddle-Wheel Type Inorganic Building Units via High-Throughput Methods. *Inorg. Chem.* **2011**, *50*, 5085–5097, doi:10.1021/ic200381f.

3 Zhang, C.; Liang, Y.; Wang, Y.; He, Y.; Majeed, A.; Yang, Z.; Yao, S.; Shen, X.; Li, T.; Qin, S. Polyamidoamine dendrimer modified Ketjen Black mixed sulfur coated cathode for enhancing polysulfides adsorbability in Li-S batteries. *Ionics* **2021**, *27*, 2997–3005, doi:10.1007/s11581-021-04100-2.

4 Xing, S.; Liang, J.; Brandt, P.; Schäfer, F.; Nuhnen, A.; Heinen, T.; Boldog, I.; Möllmer, J.; Lange, M.; Weingart, O. Capture and separation of SO_2 traces in metal–organic frameworks via pre-synthetic pore environment tailoring by methyl groups. *Angew. Chem. Int. Ed.* **2021**, *60*, 17998–18005, doi:10.1002/anie.202105229.

5 Biesinger, M.C. Accessing the robustness of adventitious carbon for charge referencing (correction) purposes in XPS analysis: Insights from a multi-user facility data review. *Appl. Surf. Sci.* **2022**, *597*, 153681, doi:10.1016/j.apsusc.2022.153681.

6 Jiao, Y.; Qu, C.; Zhao, B.; Liang, Z.; Chang, H.; Kumar, S.; Zou, R.; Liu, M.; Walton, K.S. High-Performance Electrodes for a Hybrid Supercapacitor Derived from a Metal–Organic Framework/Graphene Composite. *ACS Appl. Energy Mater.* **2019**, *2*, 5029–5038, doi:10.1021/acsaem.9b00700.

7 Wu, Y.; Song, X.; Xu, S.; Chen, Y.; Oderinde, O.; Gao, L.; Wei, R.; Xiao, G. Chemical fixation of CO_2 into cyclic carbonates catalyzed by bimetal mixed MOFs: the role of the interaction between Co and Zn. *Dalton Trans.* **2020**, *49*, 312–321, doi:10.1039/C9DT04027G.

[8] Yin, L.I.; Yellin, E.; Adler, I. X-Ray Excited LMM Auger Spectra of Copper, Nickel, and Iron. *J. Appl. Phys.* **1971**, *42*, 3595–3600, doi:10.1063/1.1660775.

[9] Grosvenor, A.P.; Kobe, B.A.; Biesinger, M.C.; McIntyre, N.S. Investigation of multiplet splitting of Fe 2p XPS spectra and bonding in iron compounds. *Surf. Interface Anal.* **2004**, *36*, 1564–1574, doi:10.1002/sia.1984.

[10] Paolella, A.; Bertoni, G.; Hovington, P.; Feng, Z.; Flacau, R.; Prato, M.; Colombo, M.; Marras, S.; Manna, L.; Turner, S. Cation exchange mediated elimination of the Fe-antisites in the hydrothermal synthesis of $LiFePO_4$. *Nano Energy* **2015**, *16*, 256–267, doi:10.1016/j.nanoen.2015.06.005.

[11] Doyle, R.L.; Lyons, M.E.G. An electrochemical impedance study of the oxygen evolution reaction at hydrous iron oxide in base. *Phys. Chem. Chem. Phys.* **2013**, *15*, 5224–5237, doi:10.1039/C3CP43464H.

[12] Doyle, R.L.; Lyons, M.E.G. Kinetics and Mechanistic Aspects of the Oxygen Evolution Reaction at Hydrous Iron Oxide Films in Base. *J. Am. Chem. Soc.* **2013**, *160*, H142, doi:10.1149/2.015303jes.

[13] Doyle, R.L.; Godwin, I.J.; Brandon, M.P.; Lyons, M.E.G. Redox and electrochemical water splitting catalytic properties of hydrated metal oxide modified electrodes. *Phys. Chem. Chem. Phys.* **2013**, *15*, 13737–13783, doi:10.1039/C3CP51213D.

[14] Öztürk, S.; Moon, G.-H.; Spieß, A.; Budiyanto, E.; Roitsch, S.; Tüysüz, H.; Janiak, C. A Highly-Efficient Oxygen Evolution Electrocatalyst Derived from a Metal-Organic Framework and Ketjenblack Carbon Material. *ChemPlusChem* **2021**, *86*, 1106–1115, doi:10.1002/cplu.202100278.

[15] Sondermann, L.; Jiang, W.; Shviro, M.; Spieß, A.; Woschko, D.; Rademacher, L.; Janiak, C. Nickel-Based Metal-Organic Frameworks as Electrocatalysts for the Oxygen Evolution Reaction (OER). *Molecules* **2022**, *27*, 1241, doi:10.3390/molecules27041241.

[16] Pang, W.; Shao, B.; Tan, X.-Q.; Tang, C.; Zhang, Z.; Huang, J. Exfoliation of metal–organic frameworks into efficient single-layer metal–organic nanosheet electrocatalysts by the synergistic action of host–guest interactions and sonication. *Nanoscale* **2020**, *12*, 3623–3629, doi:10.1039/C9NR09742B.

[17] Lin, Y.; Wan, H.; Wu, D.; Chen, G.; Zhang, N.; Liu, X.; Li, J.; Cao, Y.; Qiu, G.; Ma, R. Metal–organic framework hexagonal nanoplates: bottom-up synthesis, topotactic transformation, and efficient oxygen evolution reaction. *J. Am. Chem. Soc.* **2020**, *142*, 7317–7321, doi:10.1021/jacs.0c01916.

[18] Yu, M.; Moon, G.; Bill, E.; Tüysüz, H. Optimizing Ni–Fe Oxide Electrocatalysts for Oxygen Evolution Reaction by Using Hard Templating as a Toolbox. *ACS Appl. Energy Mater.* **2019**, *2*, 1199–1209, doi:10.1021/acsaem.8b01769.

[19] Anantharaj, S.; Karthik, P.E.; Kundu, S. Petal-like hierarchical array of ultrathin Ni(OH)2 nanosheets decorated with Ni(OH)2 nanoburls: a highly efficient OER electrocatalyst. *Catal. Sci. Technol.* **2017**, *7*, 882–893, doi:10.1039/C6CY02282K.

[20] Zhao, X.; Ding, X.; Xia, Y.; Jiao, X.; Chen, D. Coupling-Effect-Induced Acceleration of Electron Transfer for α-$Ni(OH)_2$ with Enhanced Oxygen Evolution Reaction Activity. *ACS Appl. Nano Mater.* **2018**, *1*, 1476–1483, doi:10.1021/acsanm.7b00360.
